# Developing a Black Carbon-Substituted Multimedia Model for Simulating the PAH Distributions in Urban Environments

**DOI:** 10.1038/s41598-017-14789-9

**Published:** 2017-11-06

**Authors:** Chunhui Wang, Shenglu Zhou, Yue He, Junxiao Wang, Fei Wang, Shaohua Wu

**Affiliations:** 1 School of Geographic and Oceanographic Sciences, Nanjing University, 163 Xianlin Road, Nanjing, 210023 China; 2 Nanjing Institute of Environmental Science, Ministry of Environmental Protection of China, 8 Jiangwangmiao Street, Nanjing, 210042 China

## Abstract

A multimedia fugacity model with spatially resolved environmental phases at an urban scale was developed. In this model, the key parameter, organic matter, was replaced with black carbon (BC) and applied to simulate the distributions of phenanthrene (Phe), pyrene (Pyr) and benzo[α]pyrene (BaP) in Nanjing, China. Based on the estimated emissions and measured inflows of air and water, the Phe, Pyr and BaP concentrations in different environment media were calculated under steady-state assumptions. The original model (OC-Model), BC-inclusive model (dual C-Model) and improved model (BC-Model) were validated by comparing observed and predicted Phe, Pyr and BaP concentrations. Our results suggested that lighter polycyclic aromatic hydrocarbons (PAHs) were more affected by BC substitution than their heavier counterparts. We advocate the utilization of sorption with BC in future multimedia fate models for lighter PAHs based on the comparison of the calculated and observed values from measured and published sources. The spatial distributions of the Phe, Pyr and BaP concentrations in all phases were rationally mapped based on the calculated concentrations from the BC-Model, indicating that soil was the dominant sink of PAHs in terrestrial systems, while sediment was the dominant sink of PAHs in aquatic systems.

## Introduction

Polycyclic aromatic hydrocarbons (PAHs) are ubiquitous environmental pollutants that are carcinogenic and mutagenic to humans and toxic to all living organisms^1,2^. The United States Environmental Protection Agency (US EPA) has identified 16 PAHs as priority pollutants, and these PAHs are also listed in the 1998 protocols on persistent organic pollutants under long-range transboundary air pollution^3^. Therefore, investigating the fate of PAHs in multimedia environments is very important to assess their ecological and human health risk. However, due to the complicacy of the chemicals and the paucity of systematic regional monitoring data for various environmental phases, predicting the fate and distribution of environmental multimedia has become an essential step in linking emission sources, environmental distribution, and human exposure^4^. Among various multimedia fate models, the fugacity model has been developed and applied to predict the fate of organic chemicals^5^.

Fugacity models are based on the fugacity concept, whereby the fugacity of a compound in a specific environmental phase is directly related to its concentration by means of the fugacity capacity of the phase^6,7^. One important assumption of fugacity models is that the bulk phases are completely mixed with the homogeneous concentrations of the modeled chemical^5,8–10^. Recently, 69 surface soil samples from Nanjing were measured for PAHs, and a fairly large variation was found among the sampling locations^1^. Thus, relatively large uncertainty resulted from ignoring the spatial variation, and no valuable information was obtained on the spatial distribution of the modeled chemicals^5^. Multimedia models with regional segments have been conducted in several studies, but few have been attempted at an urban scale^4,5,11^.

Multimedia fugacity models assume that organic matter is entirely responsible for the capacity of solids to sorb organic chemicals. However, increasing evidence has suggested that the existing organic matter partitioning paradigm is not sufficient to explain the sorption to environmental solids of hydrophobic organic contaminants that can obtain a planar configuration^12^. An increasing number of studies have indicated that black carbon (BC) leads to enhanced sorption of planar and aromatic organic compounds^13–15^. The formation process of BC is similar to PAHs, as the two compounds are both formed by fossil fuel combustion (traffic, industry, coal, and oil); part of their formation results from the combustion of biomass (forest fires and residential wood burning)^1,16^. BC consists of the combustion-derived carbon fraction, including residues, of initial fuel (char) as well as highly condensed carbonaceous products (soot)^17^, which are ubiquitous in the environment. The fraction of BC to total organic carbon (TOC) ranges from 5–18% in sediment samples around the world^18^, but most PAHs are bound to the BC fraction^19^. Generally, BC contents are approximately 1–15% of the TOC, and therefore, in several cases, BC can be expected to more strongly contribute to overall sorption than all the other organic matter constituents^16^. Prevedouros *et al*.^12^ developed a BC-inclusive multimedia model for predicting the fate of organics and found that lighter PAHs were more affected by BC inclusion than their heavier counterparts.

Based on the above analysis, this study attempted to improve the multimedia urban model (MUM) structure^20^, which included developing and evaluating a spatially resolved solution. We compared the simulated results among the original model (OC-Model), BC-inclusive model (dual C-Model)^12^, and BC-substituted model (BC-Model), which assumed that BC is entirely responsible for the capacity of solids to sorb organic chemicals, to determine the best model to simulate the environmental distribution of PAHs in Nanjing, China. Air, vegetation, water, soil, sediment, and organic film (“pure” film plus particles are covered on impervious surfaces) were included in the model^20^. The model was based on the steady-state, level III fugacity model of Mackay^21^, and the spatially distributed emissions of PAHs in the Nanjing urban areas from January 2012 – December 2013 were estimated and then used as the representative data for contemporary emissions. This study should provide a useful tool for modeling the distribution of PAHs in urban environments.

## Methods

### Study area descriptions

**Figure 1:**
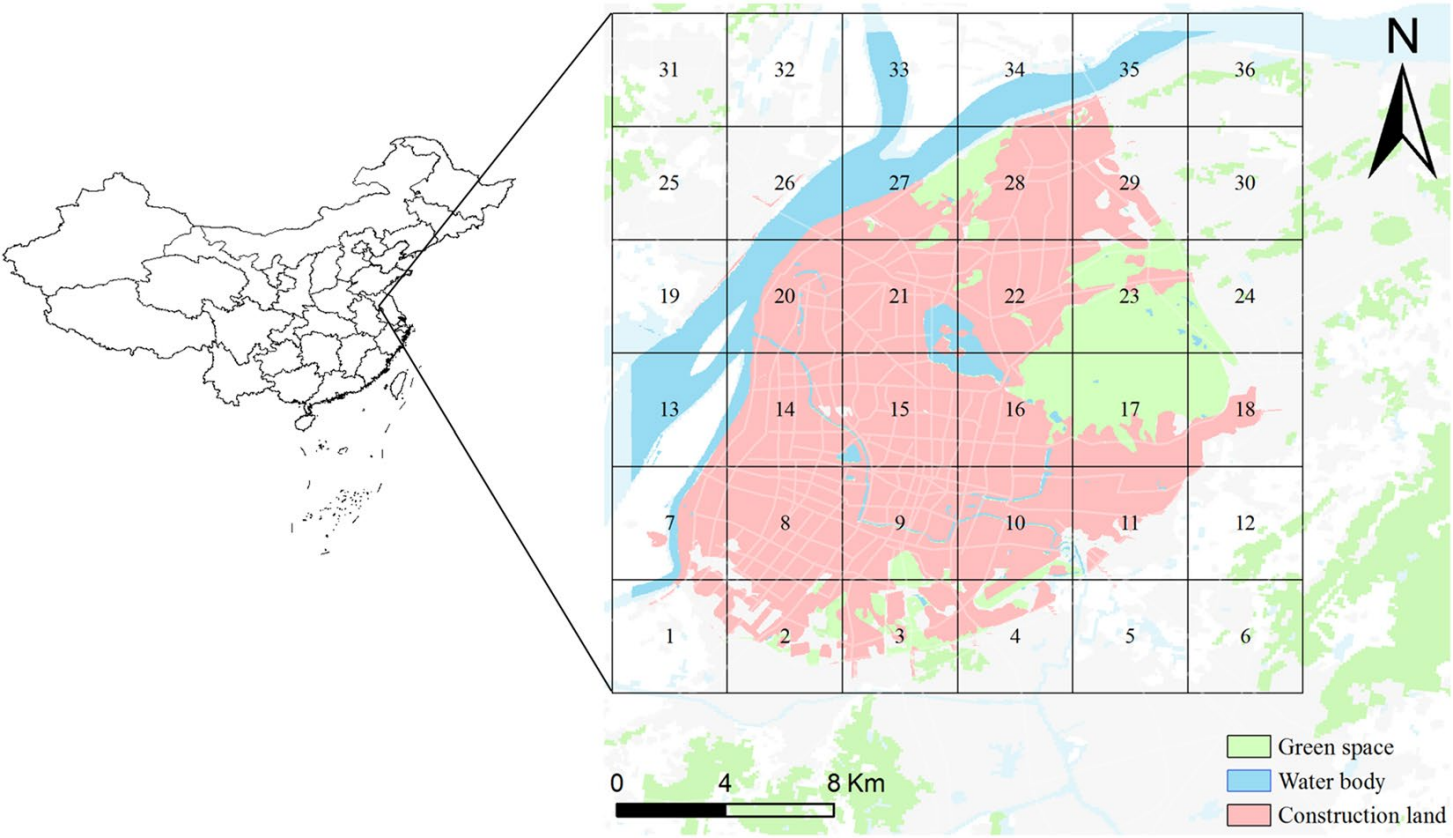
Location of the study area and model segment. (The cartographic software is ArcGIS 10.0, http://www.esri.com/).

Nanjing was selected as a prototype city. The city is an ancient capital of six dynasties with a history of more than 2500 years and a profound cultural background. As a main port along the Yangtze River, Nanjing is a complex industrial base dominated by electronics, automobile, and chemical industries. Each environmental medium in Nanjing has been heavily contaminated by many types of pollutants, including PAHs^1,22,23^. The study region was divided into 36 grid cells (4 km × 4 km). Each grid cell was constructed with six connected phases representing air, water, soil, sediment, vegetation, and organic film. Figure 1 shows the location of the study area and model segment.

### Model structure

**Figure 2:**
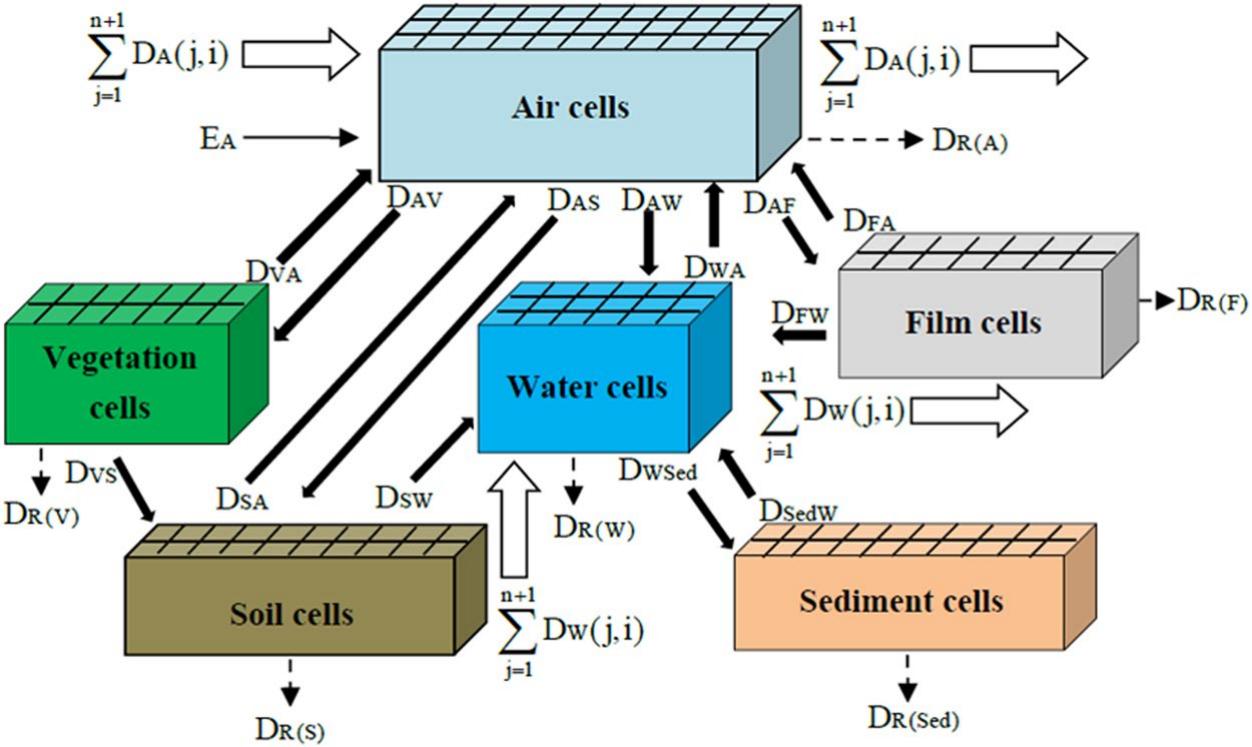
Transfer processes between the adjacent phases and phase cell. The processes are designated D_ij_. A, W, V, F, S, and Sed represent air, water, vegetation, organic film, soil and sediment media, respectively.

Like other fugacity models, three key variables, including fugacity (*f*, Pa), fugacity capacity [Z, mol/(m^3^ Pa)], and transfer rate coefficients [D, mol/(Pa h)], were employed in this model to describe the environmental distribution and fate processes. Z is specific to the properties of the contaminant and the environmental media. D represents the inter-media transport and transformation processes, including diffusion, advection, and degradation, and is related to the chemical transfer flux. The concentration of the contaminant within a phase (c, mol/m^3^) is a product of *f* and Z. The original and developed Z values for each phase are listed in the Supporting Information (Tables S1–S3). In addition, air, water, soil, sediment, vegetation, and organic film were defined as the six bulk phases for modeling the distribution of PAHs in the area. The Supporting Information (Tables S4 and S5) shows the D values and steady-state mass-balance equations used in the model. The spatial variations of air, water, soil, sediment, vegetation, and organic film were taken into consideration, and these environmental media were divided into 36 individual cells, each 4 × 4 km^2^ in area. The major chemical transfer, transformation, and fate processes are illustrated in Fig. 2. The model was applied to simulate the distribution of three chemicals, namely, phenanthrene (Phe), pyrene (Pyr) and benzo[α]pyrene (BaP), which were selected as illustrative chemicals for the wide range of PAH physical-chemical and partitioning properties.

### Parameterization

**Figure 3:**
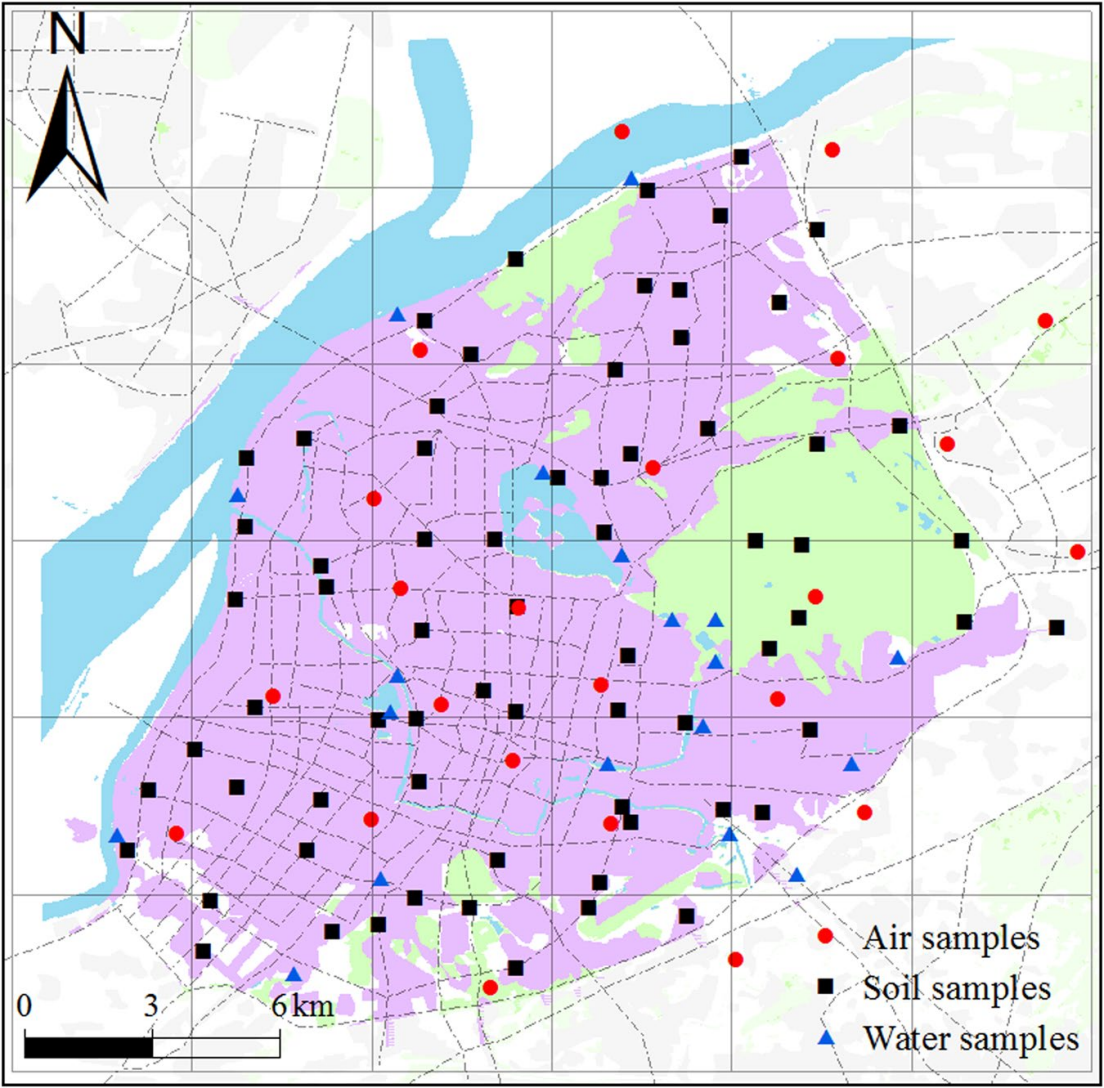
The distribution of sampling sites in the Nanjing urban areas. (The cartographic software is ArcGIS 10.0, http://www.esri.com/).

The parameters used in the model calculations include various environmental parameters, physical-chemical properties of Phe, Pyr and BaP, and flux matrixes among various media. Most of the data were obtained from the literature or from the model default value^4,24^. Information on the dimensions and areas of the various environmental media for the segments were derived from remote sensing discrimination. The physical-chemical properties of the three chemicals used as input data into this model were derived from Prevedouros *et al*.^12^, and the K_BC_ (BC-water partition coefficient) and K_BC-A_ (BC-air partition coefficient) of the three chemicals were derived from Prevedouros *et al*.^2^. The physical-chemical properties of Phe, Pyr and BaP are shown in Table S6. The rain rate and runoff rate were derived from local meteorological and hydrological data. In June 2014, 69 composite surface soil samples were collected from the Nanjing urban areas. The distribution of soil sampling sites is illustrated in Fig. 3. For each 10 m × 10 m sampling site, five soil subsamples (four corners and one center) were taken and bulked together to form one composite sample. All the samples were air-dried at room temperature for one week, sieved to 20-, 60-, and 100-mesh size particles after removing stones, residual roots, and other materials, and then stored in amber glass containers at −4 °C until analysis. The soil organic matter (SOM) contents were determined by the combustion oxidation-titration method^25^. The soil BC contents were determined by the method described by He and Zhang^26^ using a Heraeus CHN-O-Rapid elemental analyzer (GmbH, Hanau, Germany). The SOM and soil BC content results for each grid in the study area are shown in Table S7. In this research, the concentration of BC aerosol was derived from Tian *et al*.^27^. The content of BC in water suspended particulates was obtained from Huang and Zhang^28^. The concentration of BC in sediment was derived from Huang and Zhang^29^.

The air and water flows were described by matrixes of flow rates for their inter-regional movements. Airflow rates were calculated from the annual mean wind speed^30^. The external source concentrations of PAHs in the air and validation data were measured using polyurethane foam passive air samplers deployed throughout the city for a 3 month period in 2014. Twenty water samples were collected from rivers and lakes in the Nanjing urban areas to explore the external source concentrations of PAHs and to construct validation data. The advections in water and air for each grid in the study area are shown in Table S7. The distributions of the air and water sampling sites are illustrated in Fig. 3. The data for the water flow rates and water flux matrixes were collected from the Nanjing City Water Resources Bulletin^31^. Parameter values for the study area summarized in Tables S8, Table S9 and S10 contain the parameter values for chemical transfer.

### Estimated emissions

In this study, seven major sources were considered: coking production, primary aluminum production, transport petroleum combustion, non-transport petroleum combustion, industrial coal combustion, domestic coal combustion, and biomass burning. However, biomass burning, such as straw and fire wood burning, has rarely occurred in recent years in our research area. Primary aluminum is also not produced in the Nanjing urban areas, and domestic coal combustion has been replaced by coal gas and natural gas in these urban areas. Therefore, only four sources were considered in this research: transport petroleum combustion, non-transport petroleum combustion, industrial coal combustion, and coking production. The emissions from these sources were then distributed across the grid cells in the model for the gridded region.

**Figure 4:**
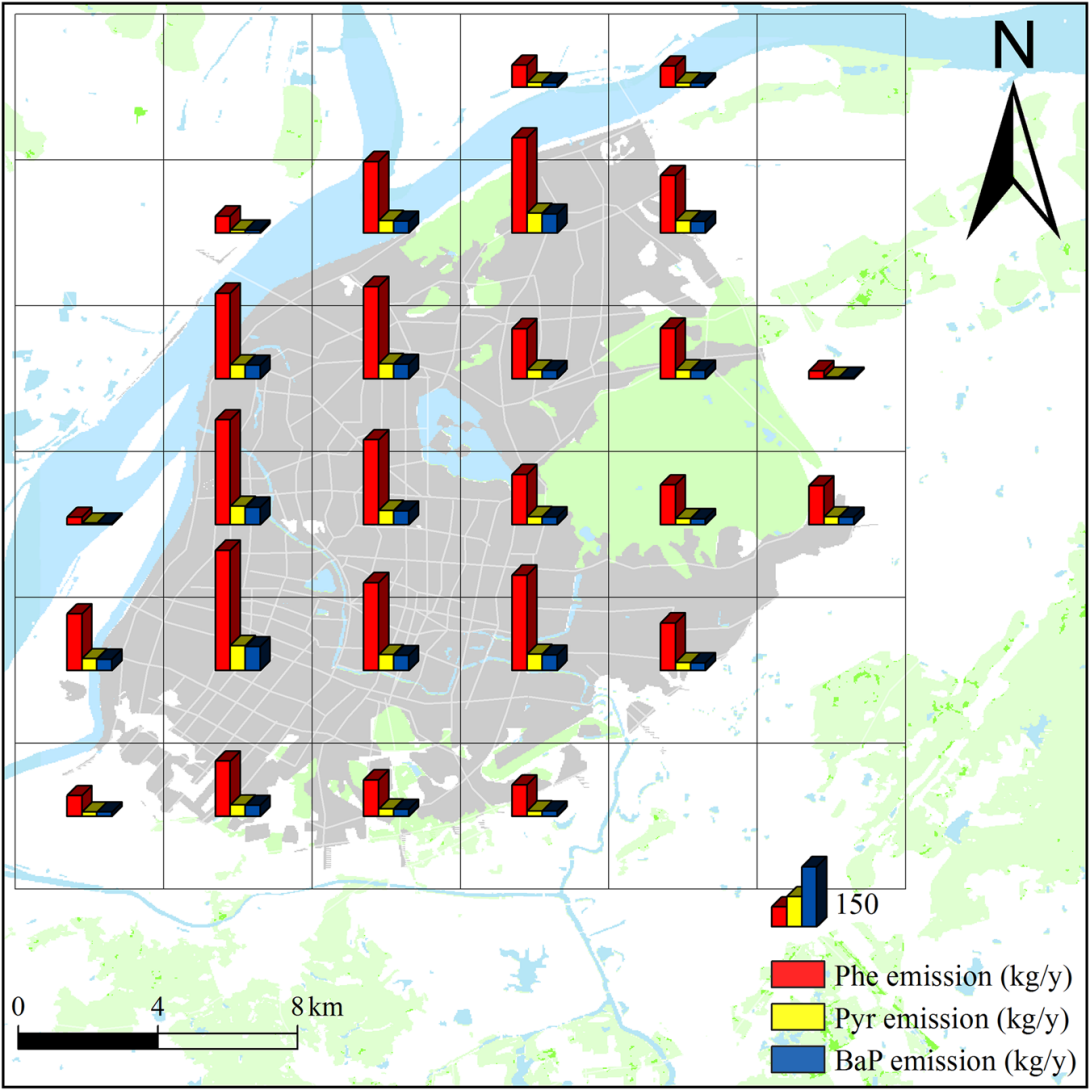
Annual average emissions of Phe, Pyr and BaP in the Nanjing urban areas from January 2012 – December 2013. (The cartographic software is ArcGIS 10.0, http://www.esri.com/).

The emission estimates in our study were based at the urban street level (known as the township level division in China), but no energy consumption data were available at this level. Zhang *et al*.^32^ found that the consumption of transport petroleum, non-transport petroleum and industrial coal linearly correlated with secondary plus tertiary GDP_s_ (GDP_23_). Energy consumption data on a provincial scale as well as data of the county and street level during January 2012 – December 2013 were collected from yearbooks^33–38^. The main coking production data were collected from official published sources^38^. Thus, the energy consumption for each street was estimated, the data were integrated into each grid using a Geographic Information System, and the emissions were calculated using the emission factors^39^. Figure 4 illustrates the spatial distribution of the Phe, Pyr and BaP emissions in the Nanjing urban areas. The total emissions of Phe, Pyr and BaP in the Nanjing urban areas were estimated as 7.05t, 1.16 and 1.22t during January 2012 – December 2013, respectively.

### Sensitivity analysis and uncertainty analysis

Sensitivity analysis is an important methodology used to test the sensitivity of the model to parameter changes and identify the most influential parameters for the model output. In this research, sensitivity analysis was performed for all the inputs parameters. Each parameter was individually adjusted by ± 10%, and the sensitivity coefficient (S) was calculated by the following formula:

1 $${S}_{k}=[{\rm{\Delta }}{Y}_{i}/{Y}_{i}]/[{\rm{\Delta }}{X}_{i}/{X}_{i}]$$

where *X*_*i*_ (*i* is the index of the input variable) denotes the input parameter of the model, Y denotes the output of the model when the test parameter is adjusted ± 10%, and Δ refers to perturbations.

Monte Carlo simulations were performed to evaluate the overall uncertainty of predictions based on the probability distributions of the input parameters. This method computes results based on repeated random sampling and statistical analysis. The model can be formulated as follows:

2 $$ < {\rm{g}} > ={\int }_{0}^{\infty }g(r)f(r)dr$$

where *g*(*r*) is the random variable, *f*(*r*) is the distribution density function, and <g> is the mathematical expectation of *g*(*r*). The approximate fo*r*mula can be converted into:

3 $${\bar{g}}_{N}=\frac{1}{N}\sum _{i=1}^{N}g({r}_{i})$$

However, most of the inputs had negligible influence on the output of the model. For this reason, all input parameters and their uncertainty were estimated according to their distribution, for which coefficients of variation were calculated to describe the distribution of the parameters based on the literature data. In this study, key input parameters were selected according to the sensitivity coefficient (S) > 0.2^37^. The values of the key input parameters were randomly selected within their respective probability distributions. The Monte Carlo simulations were repeatedly run 10000 times using Oracle Crystal Ball to obtain the distribution of the output.

## Results and Discussion

### Model Validation

Two procedures were used to test the validity of the model. One procedure assessed the calculated predictions by comparing the observations and predictions of the PAH concentrations in the bulk phase (air, water, soil, vegetation, and sediment). The other compared the prediction results among the BC-Model, dual C-Model and OC-Model.

**Figure 5:**
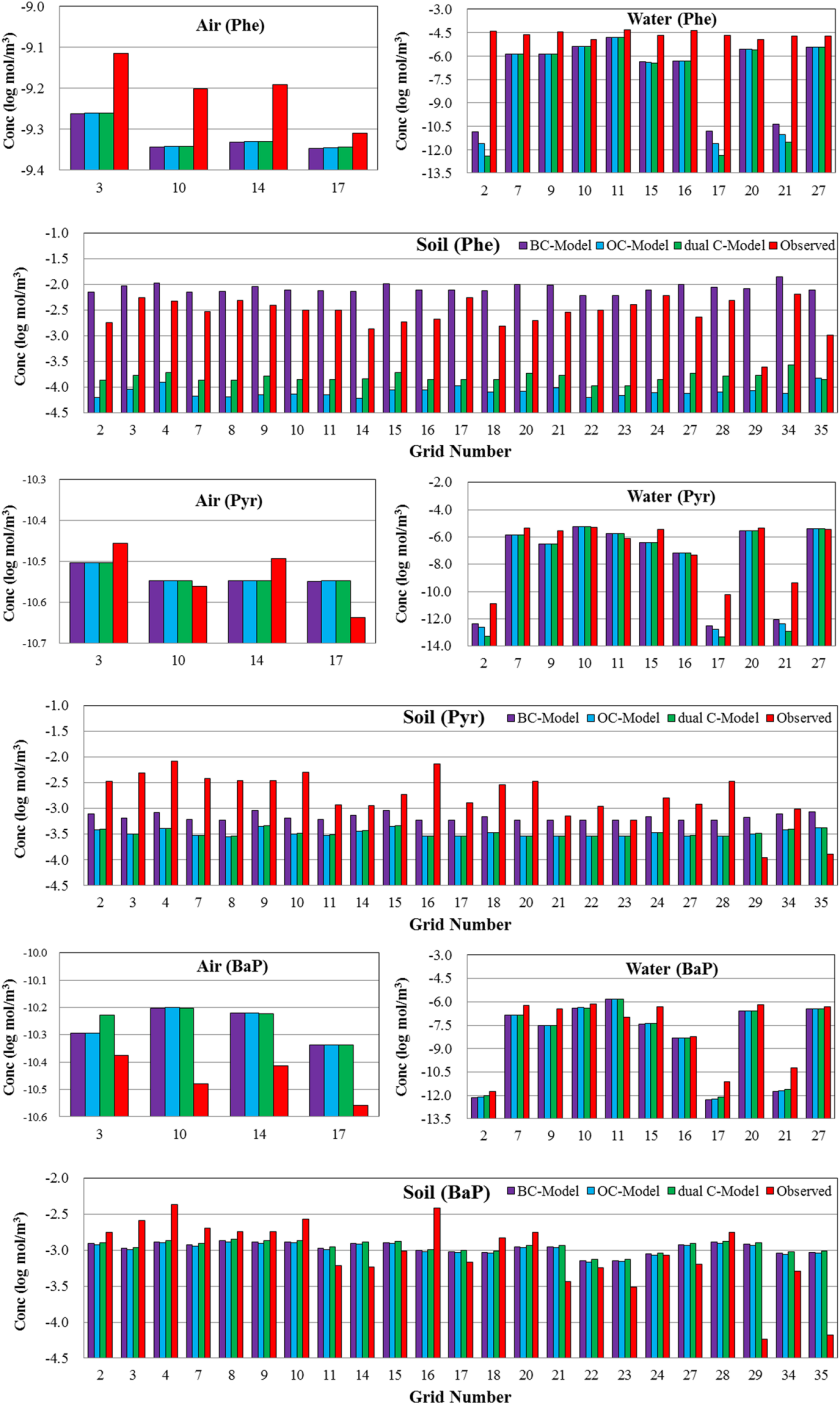
Comparison between the measured and modeled concentrations in different phases.

As shown in Fig. 5, almost no difference in the Phe concentrations in the air phase was observed among the OC-Model, dual C-Model and BC-Model. The maximum difference between the calculated and the measured concentrations was 0.14 log-units for Phe in the air phase. However, large differences in specific cells, such as 2, 17 and 21, were found for Phe in the water phase. The results could be attributed to ignore the PAHs from external water sources when simulating the fate of PAHs because of the water areas are very small in these cells. Compared to the OC-Model and dual C-Model, the soil Phe concentrations in the BC-Model were closer to the measured values. The absolute differences between the calculated and the measured mean concentrations were 0.49 log-units for the BC-Model, 1.52 log-units for the OC-Model, and 1.24 log-units for the dual C-Model. The calculated Phe concentration results in all phases for the BC-Model, dual C-Model and OC-Model are illustrated in the Supporting Information (Fig. S1); the available observed data for the Phe concentrations were collected from published data^40,41^. The independently measured mean Phe concentrations were 591.4 ng/g and 5.05 ng/g for vegetation and sediment, respectively, compared to 627.00 ng/g and 1.18 ng/g for the BC-Model, 1.63 ng/g and 0.5 ng/g for the dual C-Model, and 1.62 ng/g and 0.1 ng/g for the OC-Model, respectively. Obviously, the prediction results of the Phe concentrations in the BC-Model were closer to the observed values from the published sources. Unfortunately, measured data on organic film are not available. The calculated mean concentrations in the organic film decreased in the following order: BC-Model (−0.97 log mol/m^3^) > OC-Model (−1.67 log mol/m^3^) > dual C-Model (−2.51 log mol/m^3^).

Additionally, no difference in the Pyr concentrations in the air phase among the three models was found, and the predicted values versus the observed values were similar. In the water phase, the distribution of the calculated Pyr concentration was similar to that of the Phe concentration. In the soil phase, the calculated Pyr concentrations in the OC-Model and dual C-Model were the same but lower than the calculated value in the BC-Model, which was closer to the measured values in the most grids. However, their concentration differences fell within 1 log-unit. The calculated Pyr concentration results in the other phases are illustrated in Fig. S2. The differences of the calculated Pyr concentrations in vegetation, sediment and organic film among the three models also fell within 1 log-unit. Compared to the observed values from published data^40,41^, the absolute differences between the calculated and measured mean concentrations in vegetation fell within 1 log-unit, but the difference was greater than 1 log-unit in the sediment phase. In all cases, the difference between the modeled concentrations of the BC-Model, dual C-Model and OC-Model fell within 0.5 log-unit for BaP. Compared to the observed values, almost no difference in BaP modeled concentration was observed in the air phase. The average difference between the calculated and measured concentrations fell within 0.5 and 0.1 log-units for BaP in water and soil, respectively, which are acceptable for this type of study. The calculated BaP concentration results in the other phases are illustrated in the Supporting Information (Fig. S3). Compared to the observed values from published data^40,41^, the absolute differences between the calculated and measured mean concentrations in vegetation and sediment are both approximately 0.5 log-unit. The scatter plots of predicted versus observed values are illustrated in Fig. S4.

Based on the results of the abovementioned analysis, the BC-Model did not cause any considerable variations to the air levels and water levels of the grids containing PAHs from external water sources when simulating the fate of PAHs. However, the BC-Model caused increases in other environmental phase concentrations related to increase solid partitioning, except for BaP. In other words, the difference of the calculated concentration decreased with an increasing molecular weight of PAH in the three models. Our results are similar to the findings of Prevedouros *et al*.^42^, which indicated that lighter PAHs were more affected by BC inclusion than their heavier counterparts in all cases. In this study, we simulated the distributions and inventories of Phe, Pyr and BaP using the BC-Model, which provided more accurate simulations compared to the observed values from measured and published sources. The predicated results are as follows.

### Spatial Distribution

The spatial distributions of the Phe, Pyr and BaP concentrations in all phases were mapped based on the calculated results. As shown in Figs S5–S7, the spatial distributions of the Phe, Pyr and BaP concentrations in air, soil, vegetation, and film are very similar, and the patterns in water and sediment are alike. The higher Phe concentrations in the former group were mainly distributed in the central north-south axis, while the lowest Phe concentrations were distributed around the north of Purple Mountain (segment 23). This phenomenon could be due to traffic and population distribution in the study areas and effected by the chemical properties of Phe. The spatial distribution of Pyr was similar to that of Phe, which is attributed to their similar physical-chemistry properties. As shown in Fig. S7, the BaP concentration patterns in air, soil, vegetation, and film are very similar to the emission data, indicating that the areas at risk of BaP exposure are narrowly distributed around the emission sources. A recognizable difference in segment 4 occurred between the emission data, as shown in Fig. S7_a and S7_b, which is probably due to the influence of advective flow. Compared to the emission map, the spatial distributions of Phe, Pyr and BaP in water and sediment showed unique characteristics. The higher Phe, Pyr and BaP concentrations were mainly distributed in segments containing the Yangtze River flow, indicating that rivers are also an important risk diffusion pathway for PAHs.

### Inventory

The model predictions for the inventory in each grid are shown in Tables S11–S13. In many cases, most of the total Phe, Pyr and BaP resided in the soil, except for some segmentations mainly located in the river cells, where the total amounts of Phe, Pyr and BaP are predominantly in the sediment. Thus, soil and sediment serve as the predominant PAH sinks, as soil and sediment possessed the highest PAH retention times in the multimedia phases. In addition, the sediment and soil volumes were also important factors for their high storage capacity. The highest Phe, Pyr and BaP concentrations among all phases were in organic film, but the total amounts were low because of the small volume of organic film.

### Sensitivity and uncertainty of the model

As no differences in the source, distribution and contribution of the input parameters were observed between different segments, the sensitivity and uncertainty analysis results in one cell could be representative on the entire region^43^. Segment 27 was selected as an example for the sensitivity and uncertainty analyses. The sensitivities and uncertainties of the modeled concentrations in all the phases to the input parameters of Phe, Pyr and BaP in the OC-Model, dual C-Model and BC-Model were analyzed. The Supporting Information (Tables S14–S16) shows the absolute values of the sensitivities to the key parameters controlling the Phe, Pyr and BaP concentrations in all phases. The key parameters for the various phases were apparently different. The soil phase was affected by the highest number of key parameters in all phases, while the water was least affected. The key parameters were selected for Monte Carlo simulations according to the results of the sensitivity analysis. All the distributions of the key parameters were well fitted to log-normal distributions according to comparisons between the statistics of the fitted distribution. The results of the uncertainty analysis for the Phe, Pyr and BaP concentrations in all phases are illustrated in the Supporting Information (Tables S17–S19). The model outputs of the Phe, Pyr and BaP concentrations in soil and sediment had apparently higher uncertainties than those in the other phases due to the increased numbers of key parameters in the soil and sediment phases. Little difference of the key parameters was observed for the three chemicals among the BC-Model, dual C-Model and OC-Model. In addition, large differences of the value ranges in soil, vegetation, film and sediment phases were found for lighter PAHs in the BC-Model, dual C-Model and OC-Model, but the value ranges were similar for heavy PAHs. However, the model validation results are the same even under extreme conditions when the two extreme uncertainty values fell within 1 log-unit.

## Conclusion

In this study, a spatially resolved MUM model was developed. The key parameter, organic carbon, was displaced with BC, and model predictions were validated by comparing the observations and predictions of PAH concentrations. Our results suggested that lighter PAHs were more affected by BC substitution than their heavier counterparts. Based on the comparison of the calculated and observed values from measured and published sources, we advocate the utilization of sorption with BC in future multimedia fate models for lighter PAHs. The spatial distributions of the Phe, Pyr and BaP concentrations in all phases were rationally mapped based on the calculated concentrations of the BC-Mode, indicating that soil was the dominant sink of PAHs in terrestrial systems, while sediment was the dominant sink of PAHs in aquatic systems.

## Electronic supplementary material

Supporting information

## References

[1] Wang C (2015). Polycyclic aromatic hydrocarbons in soils from urban to rural areas in Nanjing: Concentration, source, spatial distribution, and potential human health risk. Sci Total Environ.

[2] Katsoyiannis A, Breivik K (2014). Model-based evaluation of the use of polycyclic aromatic hydrocarbons molecular diagnostic ratios as a source identification tool. Environ Pollut.

[3] UNECE. *Protocol on pesistent organic pollutants to the 1979 convention on long-range transboundary air pollution* (1998).

[4] Liu S (2014). Using gridded multimedia model to simulate spatial fate of Benzo [α] pyrene on regional scale. Environ Int.

[5] Tao S (2003). Fate modeling of phenanthrene with regional variation in Tianjin, China. Environ Sci Technol.

[6] Mackay D, Paterson S (2012). Fugacity revisited. The fugacity approach to environmental transport. Environ Sci Technol.

[7] Mackay, D. In *Multimedia environmental models: the fugacity approach*. (2nd edn) Ch. 5, 69–75 (CRC Press, 2001).

[8] Wang X (2002). Modeling the Fate of Benzo [a] pyrene in the Wastewater-Irrigated Areas of Tianjin with a Fugacity Model. J Environ Qual.

[9] Xu FL (2013). Multimedia fate modeling of polycyclic aromatic hydrocarbons (PAHs) in Lake Small Baiyangdian, Northern China. Ecol Model.

[10] Wang Z, Liu M, Yang Y, Xie Y (2011). Simulation of multimedia fate of PAHs in Shanghai City. China Environmental Science (in Chinese).

[11] Wang C (2012). A multimedia fate model to evaluate the fate of PAHs in Songhua River, China. Environ Pollut.

[12] Prevedouros K, Palm-Cousins A, Gustafsson Ö, Cousins IT (2008). Development of a black carbon-inclusive multi-media model: application for PAHs in Stockholm. Chemosphere.

[13] Dachs J, Eisenreich SJ (2000). Adsorption onto aerosol soot carbon dominates gas-particle partitioning of polycyclic aromatic hydrocarbons. Environ Sci Technol.

[14] Cornelissen G (2005). Extensive sorption of organic compounds to black carbon, coal, and kerogen in sediments and soils: mechanisms and consequences for distribution, bioaccumulation, and biodegradation. Environ Sci Technol.

[15] Accardi-Dey A, Gschwend PM (2002). Assessing the combined roles of natural organic matter and black carbon as sorbents in sediments. Environ Sci Technol.

[16] Cornelissen G, Gustafsson O (2004). Sorption of phenanthrene to environmental black carbon in sediment with and without organic matter and native sorbates. Environ Sci Technol.

[17] Goldberg, E. In *Black carbon in the environment* Ch. 1, 1–2 (Wiley, 1985).

[18] G C (2005). Extensive Sorption of Organic Compounds to Black Carbon, Coal and Kerogen in Sediments and Soils: Mechanisms and Consequences for Distribution, Bioaccumulation and Biodegradation (Critical Review). Environ Sci Technol.

[19] Oen AM, Cornelissen G, Breedveld GD (2006). Relation between PAH and black carbon contents in size fractions of Norwegian harbor sediments. Environ Pollut.

[20] Diamond ML, Priemer DA, Law NL (2001). Developing a multimedia model of chemical dynamics in an urban area. Chemosphere.

[21] Mackay D, Paterson S (1991). Evaluating the multimedia fate of organic chemicals: a level III fugacity model. Environ Sci Technol.

[22] Wu B, Zhao D, Zhang Y, Zhang X, Cheng S (2009). Multivariate statistical study of organic pollutants in Nanjing reach of Yangtze River. J Hazard Mater.

[23] He J (2014). Polycyclic aromatic hydrocarbons (PAHs) associated with fine particulate matters in Nanjing, China: distributions, sources and meteorological influences. Atmos Environ.

[24] Cao H (2003). Multimedia fate modeling with spatial resolution for phenanthrene in Tianjin. Environmental Science (in Chinese).

[25] MEPPRC. *Soil-determination of organic carbon-combustion oxidation-titration method*, http://kjs.mep.gov.cn/hjbhbz/bzwb/trhj/trjcgfffbz/201308/W020130820360848743370.pdf (2013).

[26] Yue H, Zhang G, Nanjing (2006). Concentration and sources of organic carbon and black carbon of urban soils in nanjing. Acta Pedologica Sinica (in Chinese).

[27] Tian J, Wang T, Zhuang B, Jiang A, Liu D (2013). Study on concentration and radiative forcing of black carbon aerosol in suburban Nanjing. Climatic and Environmental Research (in Chinese).

[28] Huang L, Zhang G (2014). Seasonal variations of black carbon in Xuliujing water of the Yangtze river and their environmental implications. Resources and Environment in the Yangtze Basin (in Chinese).

[29] Huang L, Zhang G (2015). Distribution of black carbon in the sediments from the Yangtze river and their correlations with polycyclic aromatic hydrocarbons. Earth and Environment (in Chinese).

[30] Geng, X. & Cao, G. Changes in wind speed and its causes in Nanjing during the period of 1960–2004. *Journal of Qinghai Meteodology (in Chinese)*, 12–18 (2014).

[31] NWCB. *Nanjing city water resources bulletin*, http://www.njsl.gov.cn/cslm/szyc/szygb/201505/P020150529632967083822.pdf (2014).

[32] Zhang Y, Tao S, Cao J, Jr CR (2007). Emission of polycyclic aromatic hydrocarbons in China by county. Environ Sci Technol.

[33] NBSC. *China energy statistical yearbook*, http://cyfd.cnki.com.cn/N2014030143.htm (2013).

[34] NBSC. *China energy statistical yearbook*, http://cyfd.cnki.com.cn/N2015110114.htm (2014).

[35] NBSC. *China statistical yearbook*, http://www.stats.gov.cn/tjsj/ndsj/2013/indexch.htm (2013).

[36] NBSC. *China statistical yearbook*, http://www.stats.gov.cn/tjsj/ndsj/2014/indexch.htm (2014).

[37] NSB. *Statistical yearbook of Nanjing*, http://221.226.86.104/file/nj2004/2013/index.htm (2013).

[38] NSB. *Statistical yearbook of Nanjing*, http://221.226.86.104/file/nj2004/2014/index.htm (2014).

[39] Xu S, Liu W, S T (2006). Emission of polycyclic aromatic hydrocarbons in China. Environ Sci Technol.

[40] Wang FW, Wang F, Yang XL, Bian YR, Jiang X (2010). Enrichment characteristics and source apportionment of polycyclic aromatic hydrocarbons (PAHs) in pine (Pinus massoniana lamb) needles from parks in Nanjing City, China. Environmental Science (in Chinese).

[41] Yang XZ (2008). Characterization and Change of Polycyclic Aromatic Hydrocarbons in Sediment from Waiqinhuai River. Research of Environmental Sciences (in Chinese).

[42] Prevedouros K, Palmcousins A, Gustafsson O, Cousins IT (2008). Development of a black carbon-inclusive multi-media model: application for PAHs in Stockholm. Chemosphere.

[43] Liu S (2013). Using gridded multimedia model to simulate spatial fate of Benzo[α]pyrene on regional scale. Environ Int.

