# Supplementary material for: Developing a Black Carbon-Substituted Multimedia Model for Simulating the PAH Distributions in Urban Environments
Source: Sci Rep. 2017 Nov 6;7:14548. doi: 10.1038/s41598-017-14789-9 (PMC5673973; doi:10.1038/s41598-017-14789-9)
Supplement: Supplementary file 1 — Supporting information [file 41598_2017_14789_MOESM1_ESM.doc]

**Supplementary Information for**

**Developing a Black Carbon-Substituted Multimedia Model for Simulating the PAH Distributions in Urban Environments**

Chunhui Wang ^1^, Shenglu Zhou ^1^,*, Yue He ^2^, Junxiao Wang ^1^, Fei Wang ^1^, Shaohua Wu ^1^,*

^1^ School of Geographic and Oceanographic Sciences, Nanjing University, 163 Xianlin Road, Nanjing, Jiangsu, 210023, PR China

^2^ State Environmental Protection Key Laboratory of Soil Environmental Management and Pollution Control, Nanjing Institute of Environmental Science, Ministry of Environmental Protection of China , Nanjing, 210042, China

**Correspondence author**:

**Name:** Shenglu Zhou; Shaohua Wu

**Address**: No.163, Xianlin Road, Qixia District, School of Geographic and Oceanographic Sciences, Nanjing University, Nanjing, Jiangsu, 210023, P.R. China

**E-mail:** zhousl@nju.edu.cn (S. Zhou); wsh@nju.edu.cn (S. Wu)

**Table S1**

Z values (mol m^-3^ Pa^-1^) calculating equations in original model (OC-model)

| **Compartment** | **Phase** | **Equation** |
| --- | --- | --- |
|  | Gas-phase | Z_A_ = 1/RT |
| Air | Particles | Z_PA_ = Z_A_ × [*f*_OM_K_OA_(ρ_Q_/1000)] |
|  | Bulk | Z_BA_ = Z_A_ + (Z_PA_ × V_PA_) |
|  |  |  |
|  | Dissolved | Z_W_ = 1/H |
| Water | Suspended Part. | Z_PW_ = Z_W_ × [ƒ_OC·PW_ K_OC_(ρ_PW_/1000)] |
|  | Bulk | Z_BW_ = Z_W_ + (Z_PW_ × V_PW_) |
|  |  |  |
| Soil | Solids | Z_S_ = Z_W_ × [ƒ_OC·S_ K_OC_(ρ_S_/1000)] |
|  | Bulk | Z_BS_ = (Z_A_ × V_A_) + (Z_W_ × V_W_) +(Z_S_ × V_S_) |
|  |  |  |
| Sediment | Solids | Z_Sed_ = Z_W_ ×[ƒ_OC·Sed_ K_OC_(ρ_Sed_/1000)] |
|  | Bulk | Z_BSed_ = (Z_W_ × V_W_) + (Z_Sed_ × V_Sed_) |
|  |  |  |
| Vegetation | Leaf cuticle | Z_V_ = Z_W_ × K_OW_ × ƒ_OC·V_ |
|  | Bulk | Z_BV_ = (Z_A_ × V_A_) + (Z_W_ × V_W_) +(Z_V_ × V_V_) |
|  |  |  |
|  | Dissolved | Z_F_ = Z_A_ × K_OA_ × ƒ_OC·F_ |
| Film | Particles | Z_PF_ = Z_A_ × [*f*_OM_K_OA_(ρ_Q_/1000)] |
|  | Bulk | Z_BF_ = (Z_F_ × Φ_F_) + (Z_PF_ ×Φ_PF_) |

Notes: A, PA, W, PW, S, Sed, V, F represent the media of air, aerosols, water, suspended sediment, soil, bulk sediment, vegetation, and organic film, respectively. B denotes the bulk Z value for that medium, H the Henry’s law constant (Pa m^3^/mol), P^S^_L_ the liquid or subcooled liquid vapor pressures (Pa), respectively, ƒ_BC_ the black carbon fraction, ρ the density (kg/l), V the volume fraction, and Φ is the mass fraction.

**Table S2**

Z values (mol m^-3^ Pa^-1^) calculating equations in dual C-model

| **Compartment** | **Phase** | **Equation** |
| --- | --- | --- |
|  | Gas-phase | Z_A_ = 1/RT |
| Air | Particles | Z_PA_ = Z_A_ [(1.5 × *f*_OC_ × K_OA_) / (ρ_OCT_ × 10^12^) + (*f*_BC_ × K_BC-A_)/10^12^] |
|  | Bulk | Z_BA_ = Z_A_ + (Z_PA_ × V_PA_) |
|  |  |  |
|  | Dissolved | Z_W_ = 1/H |
| Water | Suspended Part. | Z_PW_ = Z_W_ × ρ_PW_ × (K_OC_ *f*_OC·PW_ + K_BC_ƒ_BC·PW_[^app^C_W_/(1+DOC0.08K_OW_)]^n-1^ |
|  | Bulk | Z_BW_ = Z_W_ + (Z_PW_ × V_PW_) |
|  |  |  |
| Soil | Solids | Z_S_ = Z_W_ ×ρ_S_ × [1.5(*f*_TOC_/ρ_OCT_)K_OA_+K_BC-A_ƒ_BC·S_] |
|  | Bulk | Z_BS_ = (Z_A_ × V_A_) + (Z_W_ × V_W_) +(Z_S_ × V_S_) |
|  |  |  |
| Sediment | Solids | Z_Sed_ = Z_W_ ×ρ_Sed_ ×(K_OC_ *f*_OC·Sed_ + K_BC_ƒ_BC·Sed_Cn-1 W) |
|  | Bulk | Z_BSed_ = (Z_W_ × V_W_) + (Z_Sed_ × V_Sed_) |
|  |  |  |
| Vegetation | Leaf cuticle | Z_V_ = Z_W_ ×K_OW_ × ƒ_OC·V_ |
|  | Bulk | Z_BV_ = (Z_A_ × V_A_) + (Z_W_ × V_W_) +(Z_V_ × V_V_) |
|  |  |  |
|  | Dissolved | Z_F_ = Z_A_ × K_OA_ × ƒ_OC·F_ |
| Film | Particles | Z_PF_ = Z_A_ [(1.5 × *f*_OC_ × K_OA_) / (ρ_OCT_ × 10^12^) + (*f*_BC_ × K_BC-A_)/10^12^] |
|  | Bulk | Z_BF_ = (Z_F_ × Φ_F_) + (Z_PF_ ×Φ_PF_) |

Notes: the sediment solids – porewater partition coefficient, water particulates – water partitioning, soil – air partitioning and gas – particle partitioning are cited from [9].

**Table S3**

Z values (mol m^-3^ Pa^-1^) calculating equations in BC-model

| **Compartment** | **Phase** | **Equation** |
| --- | --- | --- |
|  | Gas-phase | Z_A_ = 1/RT |
| Air | Particles | Z_PA_ = Z_A_ × [*f*_BC_K_BC-A_(ρ_Q_/1000)] |
|  | Bulk | Z_BA_ = Z_A_ + (Z_PA_ × V_PA_) |
|  |  |  |
|  | Dissolved | Z_W_ = 1/H |
| Water | Suspended Part. | Z_PW_ = Z_W_ × [ƒ_BC·PW_K_BC_(ρ_PW_/1000)] |
|  | Bulk | Z_BW_ = Z_W_ + (Z_PW_ × V_PW_) |
|  |  |  |
| Soil | Solids | Z_S_ = Z_W_ × [ƒ_BC·S_K_BC_(ρ_S_/1000)] |
|  | Bulk | Z_BS_ = (Z_A_ × V_A_) + (Z_W_ × V_W_) +(Z_S_ × V_S_) |
|  |  |  |
| Sediment | Solids | Z_Sed_ = Z_W_ ×[ƒ_BC·Sed_ K_BC_(ρ_Sed_/1000)] |
|  | Bulk | Z_BSed_ = (Z_W_ × V_W_) + (Z_Sed_ × V_Sed_) |
|  |  |  |
| Vegetation | Leaf cuticle | Z_V_ = Z_W_ × K_BC_ × ƒ_BC·V_ |
|  | Bulk | Z_BV_ = (Z_A_ × V_A_) + (Z_W_ × V_W_) +(Z_V_ × V_V_) |
|  |  |  |
|  | Dissolved | Z_F_ = Z_A_ × K_BC-A_ × ƒ_BC·F_ |
| Film | Particles | Z_PF_ = Z_A_ × [*f*_BC_K_BC-A_(ρ_Q_/1000)] |
|  | Bulk | Z_BF_ = (Z_F_ × Φ_F_) + (Z_PF_ ×Φ_PF_) |

**Table S4**

D values (mol Pa^-1^ h^-1^) calculation equation

| **Transport: from - to** | **Process** | **Equation** |
| --- | --- | --- |
| Air - water | Gas diffusion | D_VW_ = 1/((1/K_VA_A_W_Z_A_)+(1/K_VW_A_W_Z_W_)) |
|  | Wet deposition of gas | D_RW_ = A_W_Z_W_U_R_ |
|  | Wet deposition of particles | D_PW_ = A_W_Z_PA_U_R_QV_PA_ |
|  | Dry deposition of particles | D_DW_ = A_W_Z_PA_U_P_V_PA_ |
|  | Total | D_A-W_ = D_VW_ + D_RW_ + D_PW_ + D_DW_ |
| Water - air | Gas diffusion | D_W-A_ = 1/((1/K_VA_A_W_Z_A_)+(1/K_VW_A_W_Z_W_)) |
| Air - soil | Gas diffusion | D_VS_ = 1/((1/K_AS_A_S_Z_A_)+(Y_3_/A_S_(B_A3_Z_A_+B_W3_Z_W_)))) |
|  | Wet deposition of gas | D_RS_ = A_S_Z_W_U_R_ |
|  | Wet deposition of particles | D_PS_ = A_S_Z_PA_U_R_QV_PA_ |
|  | Dry deposition of particles | D_DS_ = A_S_Z_PA_U_P_V_PA_ |
|  | Total | D_A-S_ = D_VS_ + D_RS_ + D_PS_ + D_DS_ |
| Soil - air | Gas diffusion | D_S-A_ = 1/((1/K_AS_A_S_Z_A_)+ (Y_3_/A_S_(B_A3_Z_A_+B_W3_Z_W_)))) |
| Soil - water | Soil erosion | D_SRW_ = A_S_Z_S_U_SW_ |
|  | Rainwater runoff | D_WRW_ = A_S_Z_W_U_WW_ |
|  | Total | D_S-W_ = D_SRW_ + D_WRW_ |
| Sediment - water | Diffusion | D_r_ = 1/(1/K_SedW_A_Sed_Z_W_ + Y_4_/B_W4_A_Sed_Z_W_) |
|  | Resuspension | D_RSed_ = A_Sed_Z_PSed_U_RSed_ |
|  | Total | D_Sed-W_ = D_r_ + D_RSed_ |
| Water - sediment | Diffusion | D_r_ = 1/(1/K_SedW_A_Sed_Z_W_ + Y_4_/B_W4_A_Sed_Z_W_) |
|  | Deposition | D_RSed_ = A_Sed_Z_PW_U_DP_ |
|  | Total | D_W-Sed_ = D_r_ + D_DSed_ |
| Air - film | Gas diffusion | D_VF_ = 1/((1/K_AF_A_F_Z_A_)+(1/K_FF_A_F_Z_F_)) |
|  | Wet deposition of gas | D_RF_ = A_F_Z_W_U_R_ |
|  | Wet deposition of particles | D_PF_ = A_F_Z_PA_U_R_QV_PA_ |
|  | Dry deposition of particles | D_DF_ = A_F_Z_PA_U_P_V_PA_ |
|  | Total | D_A-F_ = D_VF_ + D_RF_ + D_PF_ + D_DF_ |
| Film - air | Gas diffusion | D_F-A_ = 1/((1/K_AF_A_F_Z_A_)+(1/K_FF_A_F_Z_F_)) |
| Film - water | Rainsplash | D_F-W_ = A_F_K_FW_Z_BF_ |
| Air - vegetation | Gas diffusion | D_VV_ = 1/((1/K_AV_A_V_Z_A_)+(1/K_VV_A_V_Z_V_)) |
|  | Wet deposition of gas | D_RV_ =FrUF*U_R_A_v_Z_W_ |
|  | Wet deposition of particles | D_PV_ = FrUF*A_v_Z_PA_U_R_QV_PA_ |
|  | Dry deposition of particles | D_DV_ = A_v_Z_PA_U_P_V_PA_ |
|  | Total | D_A-V_ = D_VV_ + D_RV_ + D_PV_ + D_DV_ |
| Vegetation - air | Gas diffusion | D_V-A_ = 1/((1/K_AV_A_V_Z_A_)+(1/K_VV_A_V_Z_V_)) |
| Vegetation - soil | Canopy drip | D_CD_ = (1-FrUF)(D_RV_+D_PV_) |
|  | Wax erosion | D_WE_ = A_V_K_WE_Z_V_ |
|  | Litterfall | D_LF_ = V_V_R_LF_Z_BV_ |
|  | Total | D_V-S_ = D_CD_ + D_WE_ + D_LF_ |
| Advective processes |  | D_A(i)_ = G_(i)_Z_(i)_ |
| Degrading reactions |  | D_R(i)_ = K_(i)_V_(i)_Z_(i)_ |

Notes: A represents the media interfacial areas (m^2^), K the mass transfer coefficients (m/h), U_R_ the rain rate (m/h), U_P_ the dry deposition velocities (m/h), Q the scavenging ratio, v the volume fraction, V the medium volume, FrUF the canopy interception fractions, R_LF_ the first-order litterfall rate constants and W rainsplash rate constants.

**Table S5**

Mass balance equation

| **Media** | **Mass balance equation** |
| --- | --- |
| Air | E_A_+G_A_C_A_+D_W-A_ *f*_W_ +D_S-A_ *f*_S_ +D_V-A_ *f*_V_+D_F-A_ *f*_F_ = (D_A-W_+D_A-S_+D_A-V_+D_A-F_+D_R(A)_+D_A(A)_) *f*_A_ |
| Water | G_W_C_W_+D_A-W_*f*_A_+D_S-W_*f*_S_+D_F-W_*f*_F_+D_Sed-W_*f*_Sed_ = (D_W-A_+D_W-Sed_+D_R(W)_+D_A(W)_) *f*_W_ |
| Soil | D_A-S_*f*_A_+D_V-S_*f*_V_ = (D_S-A_+D_S-W_+D_R(S)_) *f*_S_ |
| Sediment | D_W-Sed_ *f*_W_ = (D_Sed-W_+D_R(Sed)_) *f*_Sed_ |
| Vegetation | D_A-V_f_A_ = (D_V-A_+D_V-S_+D_R(V)_)f_V_ |
| Organic film | D_A-F_f_A_ = (D_F-A_+D_F-W_+D_R(F)_) f_F_ |

**Table S6**

Physical/chemical properties of Phenanthrene, Pyrene and Benzo[α]pyrene at 25℃

| Parameters | Descriptions | Phenanthrene | Pyrene | Benzo[α]pyrene |
| --- | --- | --- | --- | --- |
| MW | Molar mass (g/mol) | 178.20 | 202.3 | 252.30 |
| VP P_S_ | Solid vapor pressure (Pa) | 0.02 | 6.00E-04 | 7.00E-07 |
| VP P_L_ | Vapor pressure of liquid (Pa) | 0.113 | 1.19E-02 | 2.10E-05 |
| lgK_BC-A_ | Black carbon-air partition coefficient | 8.8^a^ | 9.6 ^a^ | 12.4^a^ |
| lgK_BC_ | Black carbon-water partition coefficient | 5.9^a^ | 6.3 ^a^ | 8.3^a^ |
| lgK_OW_ | Octanol-water partition coefficient | 4.7 | 5.18 | 6.04 |
| lgK_OA_ | Octanol-air partition coeffieient | 7.45 | 8.61 | 10.8 |
| lgK_OC_ | Organic carbon-water coefficient | 4.15 | 4.52 | 5.74 |
| H | Henry constant (Pa m^3^/mol) | 3.24 | 0.92 | 0.046 |

a. date taken from 9.

**Table S7**

Soil TOC, soil BC and advection in air and water concentrations of urban soils in different grids

| Grids | Soil TOC (g kg^-1^) | Soil BC (g kg^-1^) | Advection in air (kg y^-1^) | | | Advection in water (kg y^-1^) | | |
| --- | --- | --- | --- | --- | --- | --- | --- | --- |
|  |  |  | Phe | Pyr | BaP | Phe | Pyr | BaP |
| 1 | 20.6 | 7.8 | 1.8E+04 | 1.7E+03 | 3.3E+03 | 5.3E+06 | 1.3E+06 | 1.2E+05 |
| 2 | 15.4 | 4.7 | 6.3E+04 | 6.5E+03 | 1.2E+04 | — | — | — |
| 3 | 21.3 | 12.6 | 8.0E+04 | 5.6E+03 | 1.1E+04 | — | — | — |
| 4 | 32.6 | 20.1 | 4.6E+04 | 3.0E+03 | 6.7E+03 | — | — | — |
| 7 | 17.6 | 4.4 | 7.9E+04 | 7.2E+03 | 1.5E+04 | 2.1E+05 | 3.3E+05 | 3.0E+04 |
| 8 | 15.8 | 6.2 | 9.6E+04 | 9.9E+03 | 2.0E+04 | — | — | — |
| 9 | 34.1 | 14.6 | 1.2E+05 | 8.7E+03 | 2.0E+04 | 2.1E+05 | 6.6E+04 | 6.0E+03 |
| 10 | 19.8 | 10.9 | 9.8E+04 | 8.7E+03 | 2.0E+04 | 6.5E+05 | 1.3E+06 | 9.2E+04 |
| 11 | 18.6 | 8.0 | 6.2E+04 | 5.4E+03 | 1.0E+04 | 2.4E+06 | 4.2E+05 | 3.4E+05 |
| 13 | 20.4 | 7.7 | 8.3E+04 | 5.5E+03 | 1.0E+04 | 7.2E+06 | 1.1E+06 | 1.0E+05 |
| 14 | 13.8 | 4.2 | 1.0E+05 | 9.1E+03 | 1.9E+04 | 1.2E+01 | 1.8E+01 | 1.6E+00 |
| 15 | 16.1 | 9.2 | 1.3E+05 | 8.5E+03 | 1.9E+04 | 5.8E+04 | 9.1E+04 | 8.3E+03 |
| 16 | 30.5 | 12.2 | 9.9E+04 | 8.0E+03 | 1.5E+04 | 7.7E+04 | 1.5E+04 | 1.1E+03 |
| 17 | 53.2 | 24.0 | 9.9E+04 | 7.8E+03 | 1.4E+04 | — | — | — |
| 18 | 23.2 | 7.4 | 5.3E+04 | 5.0E+03 | 7.6E+03 | — | — | — |
| 19 | 20.3 | 7.7 | 2.4E+04 | 1.5E+03 | 2.9E+03 | 1.9E+06 | 2.9E+05 | 2.6E+04 |
| 20 | 14.6 | 5.2 | 1.3E+05 | 8.5E+03 | 1.6E+04 | 4.1E+05 | 6.4E+05 | 5.8E+04 |
| 21 | 23.6 | 22.8 | 1.2E+05 | 8.9E+03 | 1.7E+04 | — | — | — |
| 22 | 24.0 | 19.6 | 7.6E+04 | 8.7E+03 | 1.1E+04 | — | — | — |
| 23 | 30.3 | 17.7 | 7.5E+04 | 8.7E+03 | 1.1E+04 | — | — | — |
| 24 | 21.8 | 9.7 | 1.3E+04 | 1.2E+03 | 1.8E+03 | — | — | — |
| 26 | 20.0 | 7.7 | 3.8E+04 | 2.8E+03 | 5.2E+03 | 4.2E+06 | 6.6E+05 | 6.0E+04 |
| 27 | 14.0 | 8.6 | 1.2E+05 | 7.7E+03 | 1.6E+04 | 5.8E+05 | 9.1E+05 | 8.3E+04 |
| 28 | 19.1 | 6.2 | 1.2E+05 | 9.3E+03 | 1.9E+04 | 5.8E+05 | 1.0E+05 | 9.1E+03 |
| 29 | 14.4 | 2.7 | 7.3E+04 | 5.8E+03 | 1.1E+04 | — | — | — |
| 34 | 22.6 | 5.4 | 7.3E+04 | 3.4E+03 | 5.2E+03 | 4.6E+06 | 7.2E+05 | 6.5E+04 |
| 35 | 25.6 | 12.4 | 3.2E+04 | 3.0E+03 | 4.5E+03 | 4.6E+05 | 7.1E+05 | 6.5E+04 |

**Table S8**

The environmental parameters of the study area

| Parameters | Air | Water | Soil | Sediment | Vegetation | Film |
| --- | --- | --- | --- | --- | --- | --- |
| Depth (d, m) | 1000 | 4 | 0.05 | 0.02 | 0.0002^a^ | 7.00E-08^b^ |
| Density (ρ, kg/m^3^) | 1.19 | 1000 | 1460^c^ | 1500^d^ | 850^e^ | — |
| OC/BC (f_OC_/f_BC_) | 0.2^f^/0.0588^g^ | 0.02^h^/0.0024^i^ | **TableS7** | 0.0134/0.0048^j^ | 0.02^e^/0.0022 | 0.74^b^/0.3 |
| V_A_ | 1 | — | 0.2^b^ | — | 0.18^b^ | — |
| V_W_ | — | 1 | 0.3^b^ | 0.41 | 0.8^b^ | — |
| V_PA_ | 1.04E-10 | 2.23E-04 | 0.5^b^ | 0.59 | — | 0.7^b^ |
| V_lipid_ | — | — | — | — | 0.02^b^ | 0.3^b^ |

a. date taken from 1, b. date taken from 2, c. self testing, d. date taken from 2, e. date taken from date taken from 3, f. date taken from 4, g. date taken from 5, h. date taken from 6, i. date taken from 7, j. date taken from 8.

**Table S9**

The area of environmental medium in each grid of survey region

| Grid | Area (m^2^) | | | | | |
| --- | --- | --- | --- | --- | --- | --- |
|  | Air | Water | Soil | Sediment | Vegetation | Film |
| 1 | 2.79E+06 | 4.57E+05 | 3.14E+05 | 4.57E+05 | 4.72E+05 | 7.04E+05 |
| 2 | 9.94E+06 | 9.57E+03 | 2.68E+06 | 9.57E+03 | 4.01E+06 | 3.79E+06 |
| 3 | 1.00E+07 | 5.98E+04 | 4.06E+06 | 5.98E+04 | 6.09E+06 | 4.21E+06 |
| 4 | 5.15E+06 | 8.10E+03 | 1.84E+06 | 8.10E+03 | 2.76E+06 | 2.04E+06 |
| 7 | 1.25E+07 | 1.26E+06 | 1.56E+06 | 1.26E+06 | 2.35E+06 | 3.65E+06 |
| 8 | 1.50E+07 | 1.23E+03 | 4.38E+06 | 1.23E+03 | 6.57E+06 | 1.02E+07 |
| 9 | 1.50E+07 | 3.97E+05 | 5.03E+06 | 3.97E+05 | 7.54E+06 | 9.48E+06 |
| 10 | 1.50E+07 | 6.97E+05 | 4.45E+06 | 6.97E+05 | 6.67E+06 | 9.14E+06 |
| 11 | 9.41E+06 | 1.17E+05 | 2.41E+06 | 1.17E+05 | 3.62E+06 | 5.37E+06 |
| 13 | 1.25E+07 | 7.35E+06 | 1.38E+05 | 7.35E+06 | 2.07E+05 | 3.22E+05 |
| 14 | 1.50E+07 | 1.26E+06 | 3.94E+06 | 1.26E+06 | 5.90E+06 | 9.09E+06 |
| 15 | 1.50E+07 | 7.58E+05 | 4.28E+06 | 7.58E+05 | 6.42E+06 | 9.87E+06 |
| 16 | 1.50E+07 | 1.19E+06 | 6.66E+06 | 1.19E+06 | 9.98E+06 | 7.09E+06 |
| 17 | 1.50E+07 | 1.36E+04 | 1.13E+07 | 1.36E+04 | 1.69E+07 | 3.45E+06 |
| 18 | 8.06E+06 | 1.55E+04 | 3.73E+06 | 1.55E+04 | 5.60E+06 | 1.87E+06 |
| 19 | 3.57E+06 | 2.70E+06 | 1.29E+04 | 2.70E+06 | 1.93E+04 | 3.00E+04 |
| 20 | 1.45E+07 | 4.45E+06 | 2.89E+06 | 4.45E+06 | 4.34E+06 | 6.75E+06 |
| 21 | 1.50E+07 | 1.32E+06 | 4.20E+06 | 1.32E+06 | 6.29E+06 | 9.41E+06 |
| 22 | 1.50E+07 | 6.70E+05 | 5.23E+06 | 6.70E+05 | 7.85E+06 | 9.05E+06 |
| 23 | 1.49E+07 | 8.42E+04 | 1.27E+07 | 8.42E+04 | 1.90E+07 | 1.94E+06 |
| 24 | 2.03E+06 | 1.26E+05 | 1.67E+06 | 1.26E+05 | 2.51E+06 | 4.08E+04 |
| 26 | 5.65E+06 | 3.87E+06 | 2.73E+05 | 3.87E+06 | 4.09E+05 | 6.37E+05 |
| 27 | 1.34E+07 | 5.87E+06 | 3.21E+06 | 5.87E+06 | 4.81E+06 | 3.43E+06 |
| 28 | 1.50E+07 | 6.00E+05 | 6.07E+06 | 6.00E+05 | 9.10E+06 | 7.82E+06 |
| 29 | 9.16E+06 | 7.48E+03 | 2.34E+06 | 7.48E+03 | 3.51E+06 | 4.57E+06 |
| 34 | 5.73E+06 | 4.29E+06 | 2.44E+05 | 4.29E+06 | 3.66E+05 | 5.65E+05 |
| 35 | 4.85E+06 | 3.35E+06 | 2.48E+05 | 3.35E+06 | 3.71E+05 | 5.78E+05 |

**Table S10**

Input parameters and coefficients

| Parameters | Descriptions | Value | References |
| --- | --- | --- | --- |
| K_VA_ | Air/water, air side (m/h) | 3.00E+00 | 10 |
| K_VW_ | Water/vegetation, water side (m/h) | 3.00E-02 | 10 |
| U_R_ | Rain rate (m/h) | 1.03E-04 | 11 |
| Q | Scavenging ratio | 2.00E+05 | 10 |
| U_P_ | Dry deposition velocities (m/h) | 1.08E+01 | 10 |
| K_AS_ | Soil/air, soil side (m/h) | 1.00E+00 | 10 |
| Y_3_ | Path length of molecular diffusivity in soil (m) | 5.00E-02 | 10 |
| B_A3_ | Molecular diffusivity in air (m^2^/h) | 4.00E-02 | 10 |
| B_W3_ | Molecular diffusivity in water (m^2^/h) | 4.00E-06 | 10 |
| B_W4_ | Molecular diffusivity in sediment pore water (m^2^/h) | 2.49E-06 | 12 |
| U_WW_ | Runoff rate (m/h) | 3.80E-05 | 13 |
| U_SW_ | Soil erosion rate (m/h) | 2.30E-08 | 10 |
| K_SedW_ | Water/sediment, water side (m/h) | 1.00E-02 | 10 |
| Y_4_ | Path length of molecular diffusivity in sediment (m) | 5.00E-03 | 10 |
| U_DP_ | Sediment deposition rate (m/h) | 3.88E-07 | 14 |
| U_RSed_ | Sediment resuspension rate (m/h) | 1.42E-07 | 15 |
| U_BS_ | Sediment burial rate (m/h) | 2.45E-07 | 12 |
| K_AF_ | Film/air, film side (m/h) | 4.65E+01 | 12 |
| R_LF_ | The litterfall rate constant (m/h) | 2.31E-04 | 16 |
| K_WE_ | Leaf wax erosion mass transfer coefficient (m/h) | 8.05E-08 | 17 |
| W | The film wash-off rate constant | 2.50E-01 | 17 |
| K_FW_ | Water/film, water side (m/h) | 3.50E-08 | 12 |
| K_AV_ | Vegetation/air, vegetation side (m/h) | 6.97E+01 | 12 |
| P_CV_ | Permeation rate of leaf epidermis | 2.34E-06 | 12 |
| K_VV_ | Vegetation-side mass transfer coefficient (m/h) | 4.42E+02 | 12 |
| FrUF | Canopy interception rate | 2.00E-01 | 18 |
| U_AF_ | Dry deposition velocities in leaf epidermis (m/h) | 1.50E+01 | 12 |
| P_CF_ | Film infiltration rate (m/h) | 2.34E-06 | 12 |
| K_FF_ | The film-side mass transfer coefficient (m/h) | 4.42E+02 | 12 |

**Table S11**

Percentages and concentrations of Phe amount in compartments of each grid of Nanjing

| Grid | Air | | Soil | | Water | | Sediment | | Vegetation | | Film | |
| --- | --- | --- | --- | --- | --- | --- | --- | --- | --- | --- | --- | --- |
|  | Per. | Conc. | Per. | Conc. | Per. | Conc. | Per. | Conc. | Per. | Conc. | Per. | Conc. |
| 1 | 0.2 | 7.9E-08 | 18.0 | 1.3E+00 | 9.5 | 6.0E-03 | 72.3 | 9.2E+00 | 0.0 | 4.8E-01 | 0.0 | 16.9 |
| 2 | 0.5 | 7.9E-08 | 99.3 | 1.3E+00 | 0.0 | 2.4E-09 | 0.0 | 3.7E-06 | 0.2 | 4.7E-01 | 0.0 | 16.8 |
| 3 | 0.3 | 9.7E-08 | 99.5 | 1.7E+00 | 0.0 | 3.4E-09 | 0.0 | 5.1E-06 | 0.2 | 5.8E-01 | 0.0 | 20.7 |
| 4 | 0.3 | 1.1E-07 | 99.5 | 1.9E+00 | 0.0 | 1.8E-09 | 0.0 | 2.8E-06 | 0.2 | 6.6E-01 | 0.0 | 23.3 |
| 7 | 0.9 | 7.9E-08 | 89.3 | 1.3E+00 | 1.1 | 2.4E-04 | 8.5 | 3.7E-01 | 0.2 | 4.7E-01 | 0.0 | 16.7 |
| 8 | 0.4 | 8.0E-08 | 99.4 | 1.3E+00 | 0.0 | 6.3E-09 | 0.0 | 9.6E-06 | 0.2 | 4.8E-01 | 0.0 | 16.9 |
| 9 | 0.3 | 9.5E-08 | 98.7 | 1.6E+00 | 0.1 | 2.4E-04 | 0.7 | 3.7E-01 | 0.2 | 5.7E-01 | 0.0 | 20.2 |
| 10 | 0.4 | 8.1E-08 | 94.0 | 1.4E+00 | 0.6 | 7.3E-04 | 4.8 | 1.1E+00 | 0.2 | 4.8E-01 | 0.0 | 17.2 |
| 11 | 0.4 | 8.1E-08 | 93.2 | 1.4E+00 | 0.7 | 2.7E-03 | 5.5 | 4.1E+00 | 0.2 | 4.9E-01 | 0.0 | 17.3 |
| 13 | 0.0 | 8.1E-08 | 0.5 | 1.4E+00 | 11.5 | 8.0E-03 | 88.0 | 1.2E+01 | 0.0 | 4.9E-01 | 0.0 | 17.3 |
| 14 | 0.5 | 8.3E-08 | 99.2 | 1.3E+00 | 0.0 | 6.1E-06 | 0.1 | 9.4E-03 | 0.2 | 5.0E-01 | 0.0 | 17.7 |
| 15 | 0.4 | 1.1E-07 | 98.9 | 1.9E+00 | 0.1 | 7.4E-05 | 0.4 | 1.1E-01 | 0.2 | 6.6E-01 | 0.0 | 23.3 |
| 16 | 0.3 | 8.1E-08 | 98.8 | 1.4E+00 | 0.1 | 8.7E-05 | 0.7 | 1.3E-01 | 0.2 | 4.8E-01 | 0.0 | 17.1 |
| 17 | 0.2 | 8.0E-08 | 99.6 | 1.4E+00 | 0.0 | 2.8E-09 | 0.0 | 4.3E-06 | 0.2 | 4.8E-01 | 0.0 | 17.1 |
| 18 | 0.3 | 8.2E-08 | 99.5 | 1.4E+00 | 0.0 | 1.4E-09 | 0.0 | 2.1E-06 | 0.2 | 4.9E-01 | 0.0 | 17.4 |
| 19 | 0.1 | 8.1E-08 | 0.4 | 1.4E+00 | 11.5 | 2.1E-03 | 87.9 | 3.2E+00 | 0.0 | 4.9E-01 | 0.0 | 17.3 |
| 20 | 0.5 | 1.1E-07 | 77.6 | 1.8E+00 | 2.5 | 4.7E-04 | 19.2 | 7.1E-01 | 0.2 | 6.5E-01 | 0.0 | 23.2 |
| 21 | 0.4 | 9.8E-08 | 99.4 | 1.7E+00 | 0.0 | 7.5E-09 | 0.0 | 1.1E-05 | 0.2 | 5.9E-01 | 0.0 | 20.9 |
| 22 | 0.3 | 6.2E-08 | 99.5 | 1.1E+00 | 0.0 | 4.5E-09 | 0.0 | 6.9E-06 | 0.2 | 3.7E-01 | 0.0 | 13.3 |
| 23 | 0.1 | 6.2E-08 | 99.7 | 1.1E+00 | 0.0 | 1.6E-09 | 0.0 | 2.4E-06 | 0.2 | 3.7E-01 | 0.0 | 13.2 |
| 24 | 0.1 | 8.1E-08 | 99.6 | 1.4E+00 | 0.0 | 1.7E-10 | 0.0 | 2.6E-07 | 0.2 | 4.9E-01 | 0.0 | 17.3 |
| 26 | 0.1 | 8.2E-08 | 2.9 | 1.4E+00 | 11.2 | 4.7E-03 | 85.8 | 7.2E+00 | 0.0 | 4.9E-01 | 0.0 | 17.4 |
| 27 | 0.3 | 1.1E-07 | 68.0 | 1.8E+00 | 3.6 | 6.6E-04 | 27.8 | 1.0E+00 | 0.1 | 6.4E-01 | 0.0 | 22.7 |
| 28 | 0.3 | 9.6E-08 | 96.7 | 1.6E+00 | 0.3 | 6.6E-04 | 2.4 | 1.0E+00 | 0.2 | 5.8E-01 | 0.0 | 20.4 |
| 29 | 0.5 | 9.8E-08 | 99.2 | 1.5E+00 | 0.0 | 3.5E-09 | 0.0 | 5.4E-06 | 0.2 | 5.9E-01 | 0.0 | 20.9 |
| 34 | 0.1 | 1.6E-07 | 3.9 | 2.5E+00 | 11.1 | 5.2E-03 | 84.9 | 7.9E+00 | 0.0 | 9.3E-01 | 0.0 | 33.0 |
| 35 | 0.5 | 8.2E-08 | 22.5 | 1.4E+00 | 8.9 | 5.1E-04 | 68.0 | 7.8E-01 | 0.0 | 4.9E-01 | 0.0 | 17.4 |

Note: Per. represents percentage (unit: %); Conc. represents concentration (unit: g/m^3^). The same below.

**Table S12**

Percentages and concentrations of Pyr amount in compartments of each grid of Nanjing

| Grid | Air | | Soil | | Water | | Sediment | | Vegetation | | Film | |
| --- | --- | --- | --- | --- | --- | --- | --- | --- | --- | --- | --- | --- |
|  | Per. | Conc. | Per. | Conc. | Per. | Conc. | Per. | Conc. | Per. | Conc. | Per. | Conc. |
| 1 | 0.3 | 7.9E-09 | 34.5 | 1.6E-01 | 3.5 | 1.4E-04 | 61.7 | 5.0E-01 | 0.0 | 1.5E-02 | 0.0 | 6.9E+00 |
| 2 | 0.4 | 9.7E-09 | 99.6 | 2.0E-01 | 0.0 | 1.1E-10 | 0.0 | 4.0E-07 | 0.1 | 1.8E-02 | 0.0 | 8.5E+00 |
| 3 | 0.2 | 7.9E-09 | 99.7 | 1.6E-01 | 0.0 | 1.0E-10 | 0.0 | 3.7E-07 | 0.1 | 1.5E-02 | 0.0 | 6.9E+00 |
| 4 | 0.3 | 1.0E-08 | 99.7 | 2.1E-01 | 0.0 | 6.4E-11 | 0.0 | 2.2E-07 | 0.1 | 1.9E-02 | 0.0 | 8.8E+00 |
| 7 | 0.2 | 7.5E-09 | 25.7 | 1.5E-01 | 4.0 | 3.7E-04 | 70.0 | 1.3E+00 | 0.0 | 1.4E-02 | 0.0 | 6.5E+00 |
| 8 | 0.3 | 7.2E-09 | 99.6 | 1.5E-01 | 0.0 | 2.2E-10 | 0.0 | 7.8E-07 | 0.1 | 1.3E-02 | 0.0 | 6.3E+00 |
| 9 | 0.3 | 7.2E-09 | 94.1 | 1.5E-01 | 0.3 | 7.5E-05 | 5.3 | 2.6E-01 | 0.1 | 1.3E-02 | 0.0 | 6.3E+00 |
| 10 | 0.1 | 7.2E-09 | 31.1 | 1.5E-01 | 3.7 | 1.4E-03 | 65.1 | 4.9E+00 | 0.0 | 1.3E-02 | 0.0 | 6.3E+00 |
| 11 | 0.3 | 8.1E-09 | 82.7 | 1.7E-01 | 0.9 | 4.7E-04 | 16.1 | 1.7E+00 | 0.0 | 1.5E-02 | 0.0 | 7.0E+00 |
| 13 | 0.1 | 8.1E-09 | 1.6 | 1.7E-01 | 5.2 | 1.3E-04 | 93.1 | 4.5E-01 | 0.0 | 1.5E-02 | 0.0 | 7.0E+00 |
| 14 | 0.4 | 7.2E-09 | 99.5 | 1.5E-01 | 0.0 | 2.5E-07 | 0.1 | 8.6E-04 | 0.1 | 1.3E-02 | 0.0 | 6.3E+00 |
| 15 | 0.3 | 7.2E-09 | 84.4 | 1.5E-01 | 0.8 | 1.0E-04 | 14.4 | 3.6E-01 | 0.0 | 1.3E-02 | 0.0 | 6.3E+00 |
| 16 | 0.2 | 7.1E-09 | 96.8 | 1.5E-01 | 0.2 | 1.7E-05 | 2.8 | 5.9E-02 | 0.1 | 1.3E-02 | 0.0 | 6.3E+00 |
| 17 | 0.1 | 7.1E-09 | 99.8 | 1.5E-01 | 0.0 | 8.2E-11 | 0.0 | 2.9E-07 | 0.1 | 1.3E-02 | 0.0 | 6.2E+00 |
| 18 | 0.2 | 8.5E-09 | 99.7 | 1.8E-01 | 0.0 | 5.1E-11 | 0.0 | 1.8E-07 | 0.1 | 1.6E-02 | 0.0 | 7.4E+00 |
| 19 | 0.3 | 7.5E-09 | 1.3 | 1.6E-01 | 5.2 | 4.0E-05 | 93.2 | 1.4E-01 | 0.0 | 1.4E-02 | 0.0 | 6.7E+00 |
| 20 | 0.0 | 7.2E-09 | 8.4 | 1.5E-01 | 4.9 | 7.1E-04 | 86.6 | 2.5E+00 | 0.0 | 1.4E-02 | 0.0 | 6.3E+00 |
| 21 | 0.3 | 7.2E-09 | 99.6 | 1.5E-01 | 0.0 | 2.3E-10 | 0.0 | 8.2E-07 | 0.1 | 1.3E-02 | 0.0 | 6.3E+00 |
| 22 | 0.3 | 7.2E-09 | 99.7 | 1.5E-01 | 0.0 | 2.1E-10 | 0.0 | 7.5E-07 | 0.1 | 1.3E-02 | 0.0 | 6.3E+00 |
| 23 | 0.1 | 7.2E-09 | 99.8 | 1.5E-01 | 0.0 | 5.2E-11 | 0.0 | 1.8E-07 | 0.1 | 1.3E-02 | 0.0 | 6.3E+00 |
| 24 | 0.1 | 1.7E-08 | 99.8 | 3.5E-01 | 0.0 | 1.1E-11 | 0.0 | 3.9E-08 | 0.1 | 3.2E-02 | 0.0 | 1.5E+01 |
| 26 | 0.2 | 9.0E-09 | 21.6 | 4.7E-01 | 4.1 | 7.9E-05 | 74.1 | 2.8E-01 | 0.0 | 4.8E-02 | 0.0 | 1.1E+01 |
| 27 | 0.0 | 7.3E-09 | 5.2 | 1.5E-01 | 5.1 | 1.0E-03 | 89.6 | 3.6E+00 | 0.0 | 1.4E-02 | 0.0 | 6.4E+00 |
| 28 | 0.2 | 7.2E-09 | 89.7 | 1.5E-01 | 0.5 | 1.1E-04 | 9.5 | 3.9E-01 | 0.0 | 1.3E-02 | 0.0 | 6.3E+00 |
| 29 | 0.4 | 8.1E-09 | 99.6 | 1.7E-01 | 0.0 | 1.1E-10 | 0.0 | 4.0E-07 | 0.1 | 1.5E-02 | 0.0 | 7.1E+00 |
| 34 | 0.0 | 9.6E-09 | 0.9 | 2.0E-01 | 5.3 | 8.0E-04 | 93.7 | 2.8E+00 | 0.0 | 1.8E-02 | 0.0 | 8.4E+00 |
| 35 | 0.0 | 1.0E-08 | 1.3 | 2.1E-01 | 5.3 | 8.0E-04 | 93.3 | 2.8E+00 | 0.0 | 1.9E-02 | 0.0 | 9.0E+00 |

**Table S13**

Percentages and concentrations of BaP amount in compartments of each grid of Nanjing

|  | Air | | Soil | | Water | | Sediment | | Vegetation | | Film | |
| --- | --- | --- | --- | --- | --- | --- | --- | --- | --- | --- | --- | --- |
| Grid | Per. | Conc. | Per. | Conc. | Per. | Conc. | Per. | Conc. | Per. | Conc. | Per. | Conc. |
| 1 | 0.44 | 1.44E-08 | 51.21 | 0.30 | 2.61 | 1.31E-04 | 45.70 | 4.58E-01 | 0.03 | 0.030 | 0.01 | 12.84 |
| 2 | 0.36 | 1.50E-08 | 99.57 | 0.31 | 0.00 | 1.77E-10 | 0.00 | 6.20E-07 | 0.06 | 0.031 | 0.01 | 13.22 |
| 3 | 0.24 | 1.28E-08 | 99.70 | 0.27 | 0.00 | 1.71E-10 | 0.00 | 5.99E-07 | 0.06 | 0.027 | 0.01 | 11.31 |
| 4 | 0.27 | 1.58E-08 | 99.66 | 0.33 | 0.00 | 1.01E-10 | 0.00 | 3.53E-07 | 0.06 | 0.033 | 0.01 | 13.91 |
| 7 | 0.67 | 1.44E-08 | 87.32 | 0.30 | 0.64 | 3.42E-05 | 11.30 | 1.20E-01 | 0.05 | 0.030 | 0.01 | 12.67 |
| 8 | 0.33 | 1.64E-08 | 99.60 | 0.34 | 0.00 | 5.18E-10 | 0.00 | 1.81E-06 | 0.06 | 0.034 | 0.01 | 14.52 |
| 9 | 0.29 | 1.57E-08 | 99.38 | 0.33 | 0.01 | 7.25E-06 | 0.25 | 2.55E-02 | 0.06 | 0.033 | 0.01 | 13.91 |
| 10 | 0.30 | 1.58E-08 | 92.82 | 0.33 | 0.37 | 1.03E-04 | 6.44 | 3.63E-01 | 0.06 | 0.033 | 0.01 | 13.95 |
| 11 | 0.34 | 1.29E-08 | 90.29 | 0.27 | 0.50 | 3.81E-04 | 8.80 | 1.34E+00 | 0.05 | 0.027 | 0.01 | 11.40 |
| 13 | 0.20 | 1.01E-08 | 2.25 | 0.21 | 5.26 | 1.14E-04 | 92.29 | 4.01E-01 | 0.00 | 0.021 | 0.00 | 8.94 |
| 14 | 0.37 | 1.52E-08 | 99.49 | 0.32 | 0.00 | 4.85E-07 | 0.07 | 1.70E-03 | 0.06 | 0.032 | 0.01 | 13.46 |
| 15 | 0.33 | 1.56E-08 | 98.80 | 0.32 | 0.04 | 9.78E-06 | 0.75 | 3.43E-02 | 0.06 | 0.032 | 0.01 | 13.72 |
| 16 | 0.22 | 1.19E-08 | 99.59 | 0.25 | 0.01 | 1.23E-06 | 0.12 | 4.31E-03 | 0.06 | 0.025 | 0.01 | 10.57 |
| 17 | 0.13 | 1.16E-08 | 99.81 | 0.24 | 0.00 | 1.34E-10 | 0.00 | 4.72E-07 | 0.06 | 0.024 | 0.00 | 10.22 |
| 18 | 0.21 | 1.14E-08 | 99.73 | 0.24 | 0.00 | 6.95E-11 | 0.00 | 2.45E-07 | 0.06 | 0.024 | 0.00 | 10.08 |
| 19 | 0.57 | 9.72E-09 | 2.12 | 0.20 | 5.22 | 2.96E-05 | 92.09 | 1.04E-01 | 0.00 | 0.020 | 0.00 | 8.60 |
| 20 | 0.31 | 1.35E-08 | 64.97 | 0.28 | 1.87 | 6.56E-05 | 32.80 | 2.30E-01 | 0.04 | 0.028 | 0.01 | 11.95 |
| 21 | 0.34 | 1.36E-08 | 99.58 | 0.28 | 0.00 | 4.49E-10 | 0.00 | 1.58E-06 | 0.06 | 0.029 | 0.01 | 12.05 |
| 22 | 0.28 | 8.68E-09 | 99.65 | 0.18 | 0.00 | 2.62E-10 | 0.00 | 9.19E-07 | 0.06 | 0.018 | 0.01 | 7.69 |
| 23 | 0.11 | 8.73E-09 | 99.83 | 0.18 | 0.00 | 6.43E-11 | 0.00 | 2.26E-07 | 0.06 | 0.018 | 0.00 | 7.73 |
| 24 | 0.12 | 1.07E-08 | 99.82 | 0.22 | 0.00 | 7.04E-12 | 0.00 | 2.47E-08 | 0.06 | 0.022 | 0.00 | 9.43 |
| 26 | 0.28 | 1.12E-08 | 14.02 | 0.23 | 4.62 | 6.71E-05 | 81.07 | 2.36E-01 | 0.01 | 0.023 | 0.00 | 9.87 |
| 27 | 0.22 | 1.45E-08 | 54.19 | 0.30 | 2.45 | 9.30E-05 | 43.10 | 3.27E-01 | 0.03 | 0.030 | 0.00 | 12.76 |
| 28 | 0.24 | 1.57E-08 | 98.79 | 0.33 | 0.03 | 1.06E-05 | 0.88 | 7.32E-02 | 0.06 | 0.033 | 0.01 | 13.95 |
| 29 | 0.38 | 1.47E-08 | 99.55 | 0.30 | 0.00 | 2.08E-10 | 0.00 | 7.31E-07 | 0.06 | 0.031 | 0.01 | 12.99 |
| 34 | 0.24 | 1.10E-08 | 10.61 | 0.23 | 4.81 | 7.33E-05 | 84.33 | 2.57E-01 | 0.01 | 0.023 | 0.00 | 9.72 |
| 35 | 0.26 | 1.13E-08 | 13.80 | 0.23 | 4.63 | 7.27E-05 | 81.29 | 2.55E-01 | 0.01 | 0.024 | 0.00 | 10.02 |

**Table S14**

**Sensitivity of the key parameters of the models for Phe.**

|  | Key parameters | |S_A_| | |S_S_| | |S_V_| | |S_F_| | |S_W_| | |S_Sed_| |
| --- | --- | --- | --- | --- | --- | --- | --- |
| Phe-OC Model | G_A_C_A_（W_AS_） | 0.998 | 0.998 | 0.998 | 0.998 | — | — |
|  | A_A_ | 0.909 | 0.909 | 0.909 | 0.909 | — | — |
|  | K_OW_ | — | 0.713 | 0.960 | — | — | 1.097 |
|  | V_PA_-S | — | 0.586 | — | — | — | — |
|  | ρ_S_ | — | 0.385 | — | — | — | — |
|  | d_S_ | — | 0.376 | — | — | — | — |
|  | T_1/2_-S | — | 0.368 | — | — | — | — |
|  | U_R_ | — | 0.333 | — | — | — | — |
|  | A_V_ | — | 0.309 | — | — | — | — |
|  | K_WE_ | — | 0.270 | — | — | — | — |
|  | ρ_V_ | — | — | 0.781 | — | — | — |
|  | K_OA_ | — | — | — | 0.963 | — | — |
|  | G_W_C_W_ | — | — | — | — | 1.000 | 1.000 |
|  | V_PA_-Sed | — | — | — | — | — | 0.886 |
|  | U_DP_ | — | — | — | — | — | 0.552 |
|  | B_W4_ | — | — | — | — | — | 0.308 |
| Phe-BC Model | G_A_C_A_（W_AS_） | 0.998 | 0.998 | 0.998 | 0.998 | — | — |
|  | A_A_ | 0.906 | 0.906 | 0.906 | 0.906 | — | — |
|  | A_V_ | — | 0.979 | — | — | — | — |
|  | T_1/2_-S | — | 0.976 | — | — | — | — |
|  | d_S_ | — | 0.891 | — | — | — | — |
|  | U_R_ | — | 0.527 | — | 0.615 | — | — |
|  | ρ_S_ | — | 0.909 | — | — | — | — |
|  | K_AV_ | — | 0.673 | — | — | — | — |
|  | K_WE_ | — | 0.607 | 0.336 | —— | — | — |
|  | Q | — | 0.527 | — | 0.615 | — | — |
|  | ρ_V_ | — | — | 0.909 | — | — | — |
|  | G_W_C_W_ | — | — | — | — | 1.000 | 1.000 |
|  | U_DP_ | — | — | — | — | — | 0.70 |
|  | V_PA_-Sed | — | — | — | — | — | 0.578 |
|  | ρ_Sed_ | — | — | — | — | — | 0.445 |
|  | d_Sed_ | — | — | — | — | —— | 0.383 |
|  | U_RSed_ | — | — | — | — | — | 0.367 |
| Phe-dual C Model | G_A_C_A_（W_AS_） | 0.998 | 0.998 | 0.998 | 0.998 | — | — |
|  | A_A_ | 0.909 | 0.909 | 0.909 | 0.909 | — | — |
|  | T_1/2_-S | — | 0.976 | — | — | — | — |
|  | d_S_ | — | 0.891 | — | — | — | — |
|  | ρ_S_ | — | 0.909 | — | — | — | — |
|  | K_AS_ | — | 0.312 | — | — | — | — |
|  | Y_3_ | — | 0.364 | — | — | — | — |

**Table S14 (continues)**

|  | Key parameters | |S_A_| | |S_S_| | |S_V_| | |S_F_| | |S_W_| | |S_Sed_| |
| --- | --- | --- | --- | --- | --- | --- | --- |
|  | B_A3_ | — | 0.342 | — | — | — | — |
|  | U_DP_ | — | — | — | — | — | 0.986 |
|  | K_OW_ | — | — | 0.960 | — | — | — |
|  | K_OA_ | — | — | — | 0.995 | — | — |
|  | ρ_V_ | — | — | 0.909 | — | — | — |
|  | G_W_C_W_ | — | — | — | — | 1.000 | 1.000 |
|  | ρ_Sed_ | — | — | — | — | — | 0.909 |
|  | U_RSed_ | — | — | — | — | — | 0.466 |
|  | V_PA_-Sed | — | — | — | — | — | 0.465 |
|  | T_1/2_-Sed | — | — | — | — | — | 0.487 |
|  | d_Sed_ | — | — | — | — | — | 0.486 |

Notes: G_A_C_A_ is the atmospheric advection input; T_1/2_-S the half-life in soil phase; d_S_ the soil depth; ρ_S_ the soil density; V_PA_-S the proportion of particulate matter in soil phase; A_A_ the atmospheric area; W_AS_ the meanannual wind speed; G_W_C_W_ the water advection input.

**Table S15**

**Sensitivity of the key parameters of the models for Pyr.**

|  | Key parameters | | |S_A_| | | |S_S_| | | |S_V_| | | |S_F_| | | |S_W_| | | |S_Sed_| | |  |
| --- | --- | --- | --- | --- | --- | --- | --- | --- | --- | --- | --- | --- | --- | --- | --- |
| Pyr-OC Model | G_A_C_A_（W_AS_） | | 0.769 | | 0.769 | | 0.769 | | 0.769 | | — | | — | |  |
|  | A_A_ | | 0.908 | | 0.908 | | 0.908 | | 0.908 | | — | | — | |  |
|  | K_OW_ | | — | | 0.481 | | 0.699 | | — | | — | | 0.829 | |  |
|  | T_1/2_-S | | — | | 0.934 | | — | | — | | — | | — | |  |
|  | d_S_ | | — | | 0.859 | | — | | — | | — | | — | |  |
|  | A_V_ | | — | | 0.687 | | — | | — | | — | | — | |  |
|  | K_WE_ | | — | | 0.536 | | — | | — | | — | | — | |  |
|  | U_R_ | | — | | 0.314 | | — | | — | | — | | — | |  |
|  | K_AV_ | | — | | — | | 0.243 | | — | | — | | — | |  |
|  | K_OA_ | | — | | — | | — | | 0.637 | | — | | — | |  |
|  | K_AF_ | | — | | — | | — | | 0.203 | | — | | — | |  |
|  | K_FW_ | | — | | — | |  | | 0.310 | | — | | — | |  |
|  | G_W_C_W_ | | — | | — | | — | | — | | 1.000 | | 1.000 | |  |
|  | A_S_ | | — | | 0.626 | | — | | — | | — | | — | |  |
|  | d_Sed_ | | — | | — | | — | | — | | — | | 0.217 | |  |
|  | V_PA_-Sed | | — | | — | | — | | — | | — | | 0.761 | |  |
|  | T_1/2_-Sed | | — | | — | | — | | — | | — | | 0.206 | |  |
|  | U_DP_ | | — | | — | | — | | — | | — | | 0.788 | |  |
|  | U_RSed_ | | — | | — | | — | | — | | — | | 0.207 | |  |
|  | ρ_S_ | | — | | 0.877 | | — | | — | | — | | — | |  |
|  | ρ_V_ | | — | | — | | 0.909 | | — | | — | | — | |  |
| Pyr-BC Model | G_A_C_A_（W_AS_） | | 0.769 | | 0.769 | | 0.769 | | 0.769 | | — | | — | |  |
|  | A_A_ | | 0.906 | | 0.906 | | 0.906 | | 0.906 | | — | | — | |  |
|  | ρ_S_ | | — | | 0.909 | | — | | — | | — | | — | |  |
|  | T_1/2_-S | | — | | 0.976 | | — | | — | | — | | — | |  |
|  | d_S_ | | — | | 0.891 | | — | | — | | — | | — | |  |
|  | A_S_ | | — | | 0.261 | | — | | — | | — | | — | |  |
|  | U_R_ | | — | | 0.548 | | — | | — | | — | | — | |  |
|  | A_V_ | | — | | 0.285 | | — | | — | | — | | — | |  |
|  | ρ_V_ | | — | | — | | 0.909 | | — | | — | | — | |  |
|  | K_FW_ | | — | | — | | — | | 0.875 | | — | | — | |  |
|  | G_W_C_W_ | | — | | — | | — | | — | | 1.000 | | 1.000 | |  |
|  | ρ_Sed_ | | — | | — | | — | | — | | — | | 0.903 | |  |
|  | U_DP_ | | — | | — | | — | | — | | — | | 0.998 | |  |
|  | d_Sed_ | | — | | — | | — | | — | | — | | 0.485 | |  |
|  | V_PA_-Sed | | — | | — | | — | | — | | — | | 0.466 | |  |
|  | T_1/2_-Sed | | — | | — | | — | | — | | — | | 0.486 | |  |
|  | U_RSed_ | | — | | — | | — | | — | | — | | 0.465 | |  |
| Pyr-dual C Model | G_A_C_A_（W_AS_） | | 0.769 | | 0.769 | | 0.769 | | 0.769 | | — | | — | |  |
|  | K_OW_ | | — | | 0.460 | | 0.698 | | — | | — | | — | |  |
|  | K_OA_ | | — | | — | | — | | 0.928 | | — | | — | |  |
|  | T_1/2_-S | | — | | 0.976 | | — | | — | | — | | — | |  |
|  | | A_A_ | | 0.908 | | 0.908 | | 0.908 | | 0.908 | | — | | — | |
|  | | G_W_C_W_ | | — | | — | | — | | — | | 1.000 | | 1.000 | |

**Table S15 (continues)**

|  | | Key parameters | | |S_A_| | | |S_S_| | | |S_V_| | | |S_F_| | | |S_W_| | | |S_Sed_| | |
| --- | --- | --- | --- | --- | --- | --- | --- | --- | --- | --- | --- | --- | --- | --- | --- |
|  | d_S_ | | — | | 0.891 | | — | | — | | — | | — | |  |
|  | | U_R_ | | — | | 0.301 | | — | | — | | — | | — | |
|  | | A_S_ | | — | | 0.644 | | — | | — | | — | | — | |
|  | | A_V_ | | — | | 0.707 | | — | | — | | — | | — | |
|  | | ρ_Sed_ | | — | | — | | — | | — | | — | | 0.909 | |
|  | | d_Sed_ | | — | | — | | — | | — | | — | | 0.487 | |
|  | | V_PA_-Sed | | — | | — | | — | | — | | — | | 0.465  465 | |
|  | | T_1/2_-Sed | | — | | — | | — | | — | | — | | 0.488 | |
|  | | U_DP_ | | — | | — | | — | | — | | — | | 0.779 | |
|  | | U_RSed_ | | — | | — | | — | | — | | — | | 0.466 | |
|  | | B_W4_ | | — | | — | | — | | — | | — | | 0.209 | |
|  | | K_BC_ | | — | | — | | — | | — | | — | | 0.273 | |
|  | | ρ_V_ | | — | | — | | 0.909 | | — | | — | | — | |

**Table S16**

Sensitivity of the key parameters of the models for BaP.

|  | Key parameters | |S_A_| | |S_S_| | |S_V_| | |S_F_| | |S_W_| | |S_Sed_| |
| --- | --- | --- | --- | --- | --- | --- | --- |
| BaP-OC Model | G_A_C_A_（W_AS_） | 0.998 | 0.998 | 0.998 | 0.998 | — | — |
|  | A_A_ | 0.906 | 0.906 | 0.906 | 0.906 | — | — |
|  | T_1/2_-S | — | 0.970 | — | — | — | — |
|  | d_S_ | — | 0.886 | — | — | — | — |
|  | U_R_ | — | 0.476 | — | — | — | — |
|  | ρ_S_ | — | 0.905 | — | — | — | — |
|  | Q | — | 0.440 | — | 0.440 | — | — |
|  | A_V_ | — | 0.394 | — | — | — | — |
|  | A_S_ | — | 0.359 | — | — | — | — |
|  | ρ_V_ | — | — | 0.909 | — | — | — |
|  | K_OW_ | — | — | 0.279 | — | — | 0.219 |
|  | K_OA_ | — | — | 0.372 | — | — | — |
|  | K_AV_ | — | — | 0.324 | — | — | — |
|  | K_WE_ | — | — | 0.332 | — | — | — |
|  | G_W_C_W_ | — | — | — | — | 1.000 | 1.000 |
|  | U_DP_ | — | — | — | — | — | 0.964 |
|  | V_PA_-Sed | — | — | — | — | — | 0.543 |
|  | ρ_Sed_ | — | — | — | — | — | 0.710 |
|  | d_Sed_ | — | — | — | — | — | 0.415 |
|  | U_RSed_ | — | — | — | — | — | 0.397 |
| BaP-BC Model | G_A_C_A_（W_AS_） | 0.998 | 0.998 | 0.998 | 0.998 | — | — |
|  | A_A_ | 0.906 | 0.906 | 0.906 | 0.906 | — | — |
|  | T_1/2_-S | — | 0.976 | — | — | — | — |
|  | d_S_ | — | 0.891 | — | — | — | — |
|  | U_R_ | — | 0.529 | 0.229 | — | — | — |
|  | ρ_S_ | — | 0.909 | — | — | — | — |
|  | A_S_ | — | 0.271 | — | — | — | — |
|  | A_V_ | — | 0.296 | — | — | — | — |
|  | Q | — | 0.523 | — | — | — | — |
|  | ρ_V_ | — | — | 0.909 | — | — | — |
|  | K_WE_ | — | — | 0.467 | — | — | — |
|  | G_W_C_W_ | — | — | — | — | 1.000 | 1.000 |
|  | U_DP_ | — | — | — | — | — | 0.998 |
|  | V_PA_-Sed | — | — | — | — | — | 0.465 |
|  | ρ_Sed_ | — | — | — | — | — | 0.904 |
|  | d_Sed_ | — | — | — | — | — | 0.486 |
|  | U_RSed_ | — | — | — | — | — | 0.465 |
| BaP-dual C Model | G_A_C_A_（W_AS_） | 0.812 | 0.812 | 0.812 | 0.812 | — | — |
|  | A_A_ | 0.906 | 0.906 | 0.906 | 0.906 | — | — |
|  | K_OW_ | — | 0.211 | 0.252 | — | — | — |

**Table S16 (continues)**

|  | Key parameters | |S_A_| | |S_S_| | |S_V_| | |S_F_| | |S_W_| | |S_Sed_| |
| --- | --- | --- | --- | --- | --- | --- | --- |
|  | T_1/2_-S | — | 0.976 | — | — | — | — |
|  | d_S_ | — | 0.891 | — | — | — | — |
|  | ρ_S_ | — | 0.909 | — | — | — | — |
|  | ρ_V_ | — | — | 0.909 | — | — | — |
|  | G_W_C_W_ | — | — | — | — | 1.000 | 1.000 |
|  | U_DP_ | — | — | — | — | — | 0.998 |
|  | ρ_Sed_ | — | — | — | — | — | 0.909 |
|  | U_RSed_ | — | — | — | — | — | 0.466 |
|  | V_PA_-Sed | — | — | — | — | — | 0.465 |
|  | T_1/2_-Sed | — | — | — | — | — | 0.488 |
|  | d_Sed_ | — | — | — | — | — | 0.487 |

Notes: A_V_ the vegetation area; A_S_ the soil area

**Table S17**

Uncertainty analysis of the model for Phe.

| Environmental medium | CV | Mean | Min | Max | SD |
| --- | --- | --- | --- | --- | --- |
| Phe-OC Model air（ng/m^3^） | 0.1418 | 108.15 | 63.28 | 186.61 | 15.33 |
| Phe-OC Model soil（ng/g） | 0.1867 | 9.94 | 4.79 | 22.52 | 1.86 |
| Phe-OC Model vegetation（ng/g） | 0.1965 | 1.99 | 0.92 | 3.91 | 0.39 |
| Phe-OC Model organic film（g/m^3^） | 0.1695 | 4.60 | 2.37 | 8.77 | 0.78 |
| Phe-OC Model water（ng/l） | 0.1004 | 653.48 | 409.59 | 956.21 | 65.61 |
| Phe-OC Model sediment（ng/g） | 0.1855 | 141.93 | 68.53 | 319.01 | 26.32 |
| Phe-BC Model air（ng/m^3^） | 0.1412 | 107.98 | 61.81 | 184.08 | 15.25 |
| Phe-BC Model soil（ng/g） | 0.2571 | 1256.05 | 426.85 | 3055.09 | 322.87 |
| Phe-BC Model vegetation（ng/g） | 0.1795 | 769.38 | 389.69 | 1472.28 | 138.11 |
| Phe-BC Model organic film（g/m^3^） | 0.1396 | 23.07 | 13.77 | 38.68 | 3.22 |
| Phe-BC Model water（ng/l） | 0.1008 | 654.23 | 432.82 | 948.14 | 65.91 |
| Phe-BC Model sediment（ng/g） | 0.1546 | 669.45 | 379.91 | 1201.9 | 103.47 |
| Phe-dual C Model air（ng/m^3^） | 0.142 | 106.75 | 61.5 | 180.7 | 15.16 |
| Phe-dual C Model soil（ng/g） | 0.2316 | 23.10 | 9.6 | 58.6 | 5.35 |
| Phe-dual C Model vegetation（ng/g） | 0.1734 | 1.96 | 0.97 | 3.68 | 0.34 |
| Phe-dual C Model organic film（g/m^3^） | 0.1768 | 0.66 | 0.34 | 1.36 | 0.12 |
| Phe-dual C Model water（ng/l） | 0.1 | 652.61 | 433.74 | 900.33 | 65.26 |
| Phe-dual C Model sediment（ng/g） | 0.1995 | 1495.13 | 741.68 | 2930.00 | 298.24 |

**Table S18**

Uncertainty analysis of the model for Pyr.

| Environmental medium | CV | Mean | Min | Max | SD |
| --- | --- | --- | --- | --- | --- |
| Pyr -OC Model air（ng/m^3^） | 0.126 | 5.96 | 3.85 | 9.27 | 0.75 |
| Pyr -OC Model soil（ng/g） | 0.2449 | 41.62 | 16.81 | 93.15 | 10.19 |
| Pyr -OC Model vegetation（ng/g） | 0.1782 | 16.30 | 8.21 | 31.68 | 2.91 |
| Pyr -OC Model organic film（g/m^3^） | 0.1488 | 2.80 | 1.49 | 4.97 | 0.42 |
| Pyr -OC Model water（ng/l） | 0.0995 | 813.46 | 559.28 | 1181.97 | 80.91 |
| Pyr -OC Model sediment（ng/g） | 0.1787 | 527.51 | 252.78 | 1024.24 | 94.26 |
| Pyr -BC Model air（ng/m^3^） | 0.1249 | 5.93 | 3.72 | 10.16 | 0.74 |
| Pyr -BC Model soil（ng/g） | 0.2232 | 86.21 | 38.87 | 218.85 | 19.24 |
| Pyr -BC Model vegetation（ng/g） | 0.1645 | 13.31 | 7.47 | 25.36 | 2.19 |
| Pyr -BC Model organic film（g/m^3^） | 0.1562 | 5.24 | 2.57 | 9.20 | 0.82 |
| Pyr -BC Model water（ng/l） | 0.0988 | 815.06 | 523.22 | 1171.87 | 80.56 |
| Pyr -BC Model sediment（ng/g） | 0.2031 | 1923.70 | 918.05 | 5206.08 | 390.70 |
| Pyr -dual C Model air（ng/m^3^） | 0.1248 | 5.96 | 3.65 | 9.02 | 0.74 |
| Pyr -dual C Model soil（ng/g） | 0.1988 | 41.89 | 18.23 | 89.80 | 8.33 |
| Pyr -dual C Model vegetation（ng/g） | 0.1777 | 16.62 | 8.28 | 32.91 | 2.95 |
| Pyr -dual C Model organic film（g/m^3^） | 0.1578 | 0.56 | 0.31 | 1.03 | 0.09 |
| Pyr -dual C Model water（ng/l） | 0.0994 | 814.19 | 538.54 | 1170.65 | 80.93 |
| Pyr -dual C Model sediment（ng/g） | 0.1946 | 1216.30 | 547.13 | 2454.65 | 236.67 |

**Table S19**

Uncertainty analysis of the model for BaP.

| Environmental medium | CV | Mean | Min | Max | SD |
| --- | --- | --- | --- | --- | --- |
| BaP-OC Model air（ng/m^3^） | 0.1405 | 12.22 | 7.4 | 20.68 | 1.72 |
| BaP-OC Model soil（ng/g） | 0.2395 | 170.46 | 69.14 | 404.06 | 40.82 |
| BaP-OC Model vegetation（ng/g） | 0.1882 | 35.07 | 17.17 | 71.94 | 6.6 |
| BaP-OC Model organic film（g/m^3^） | 0.1475 | 11.97 | 6.43 | 20.6 | 1.77 |
| BaP-OC Model water（ng/l） | 0.0993 | 92.46 | 62.62 | 141.54 | 9.18 |
| BaP-OC Model sediment（ng/g） | 0.1789 | 169.33 | 85.8 | 328.1 | 30.29 |
| BaP-BC Model air（ng/m^3^） | 0.1385 | 12.18 | 6.92 | 19.92 | 1.69 |
| BaP-BC Model soil（ng/g） | 0.2408 | 177.03 | 70.74 | 428.6 | 42.63 |
| BaP-BC Model vegetation（ng/g） | 0.1796 | 30.61 | 14.49 | 56.68 | 5.5 |
| BaP-BC Model organic film（g/m^3^） | 0.14 | 10.8 | 6.44 | 17.98 | 1.51 |
| BaP-BC Model water（ng/l） | 0.0992 | 92.55 | 62.82 | 130.94 | 9.18 |
| BaP-BC Model sediment（ng/g） | 0.1926 | 219.3 | 105.68 | 415.32 | 42.33 |
| BaP -dual C Model air（ng/m^3^） | 0.1291 | 14.68 | 8.44 | 23.93 | 1.89 |
| BaP -dual C Model soil（ng/g） | 0.2164 | 220.54 | 98.20 | 456.41 | 47.72 |
| BaP -dual C Model vegetation（ng/g） | 0.1641 | 109.36 | 56.77 | 211.54 | 17.95 |
| BaP -dual C Model organic film（g/m^3^） | 0.1286 | 19.18 | 11.32 | 31.16 | 2.47 |
| BaP -dual C Model water（ng/l） | 0.1011 | 92.57 | 62.17 | 133.82 | 9.36 |
| BaP -dual C Model sediment（ng/g） | 0.2034 | 218.97 | 104.55 | 444.10 | 44.54 |

**Figure Legends**

**Figure S1.** Comparison among BC-model, OC-model and dual C-model in different compartments for Phe.

**Figure S2.** Comparison among BC-model, OC-model and dual C-model in different compartments for Pyr.

**Figure S3.** Comparison among BC-model, OC-model and dual C-model in different compartments for BaP.

**Figure S4.** Scatter plots of predicted versus observed values in air, water and soil phases.

**Figure S5.** Modeled concentration of Phe in air (a), soil (b), water (c), sediment (d), vegetation (e) and film (f) in Nanjing urban area. (The cartographic software is ArcGIS 10.0 URL: http://www.esri.com/)

**Figure S6.** Modeled concentration of Pyr in air (a), soil (b), water (c), sediment (d), vegetation (e) and film (f) in Nanjing urban area. (The cartographic software is ArcGIS 10.0 URL: http://www.esri.com/)

**Figure S7.** Modeled concentration of BaP in air (a), soil (b), water (c), sediment (d), vegetation (e) and film (f) in Nanjing urban area. (The cartographic software is ArcGIS 10.0 URL: http://www.esri.com/)


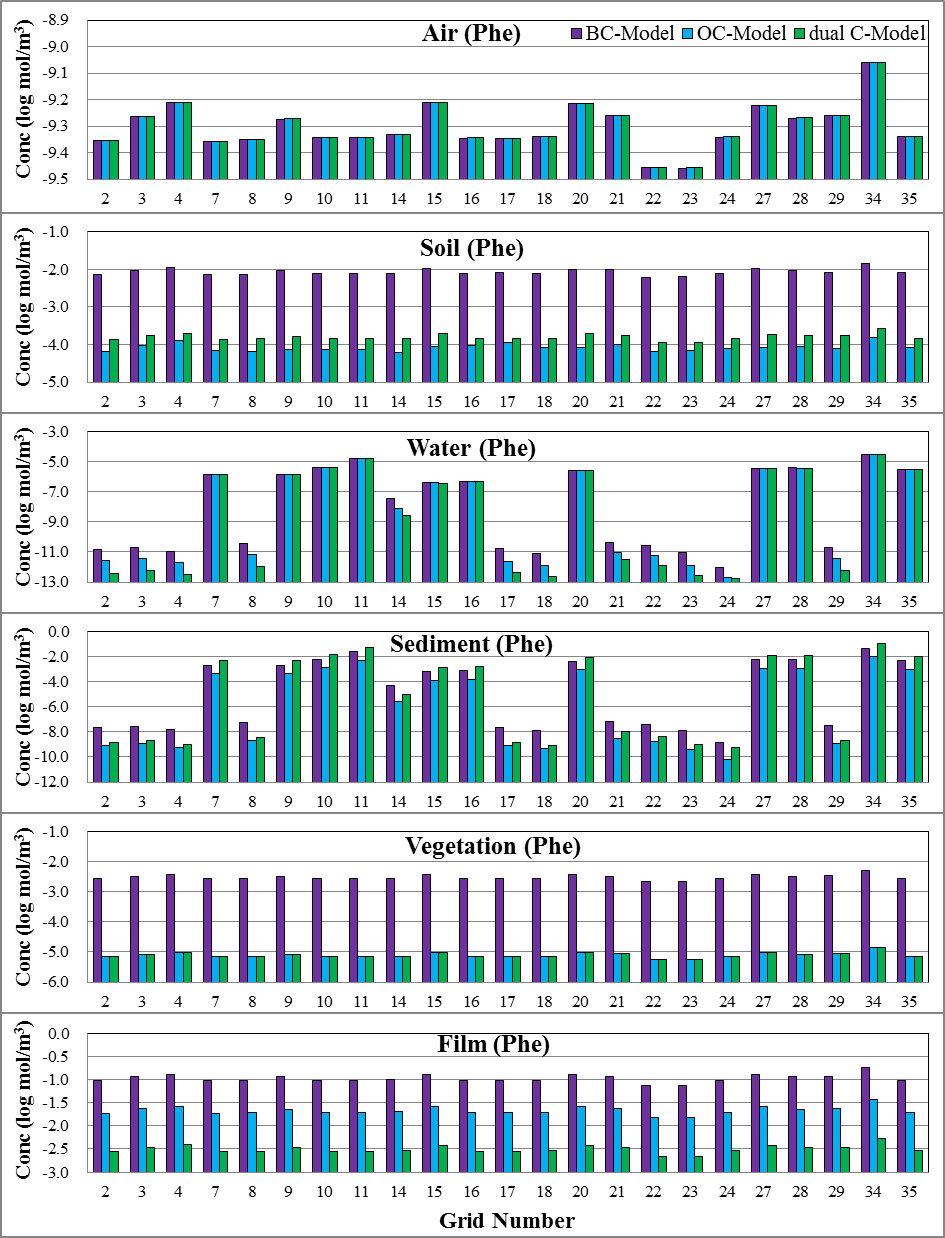


Figure S1


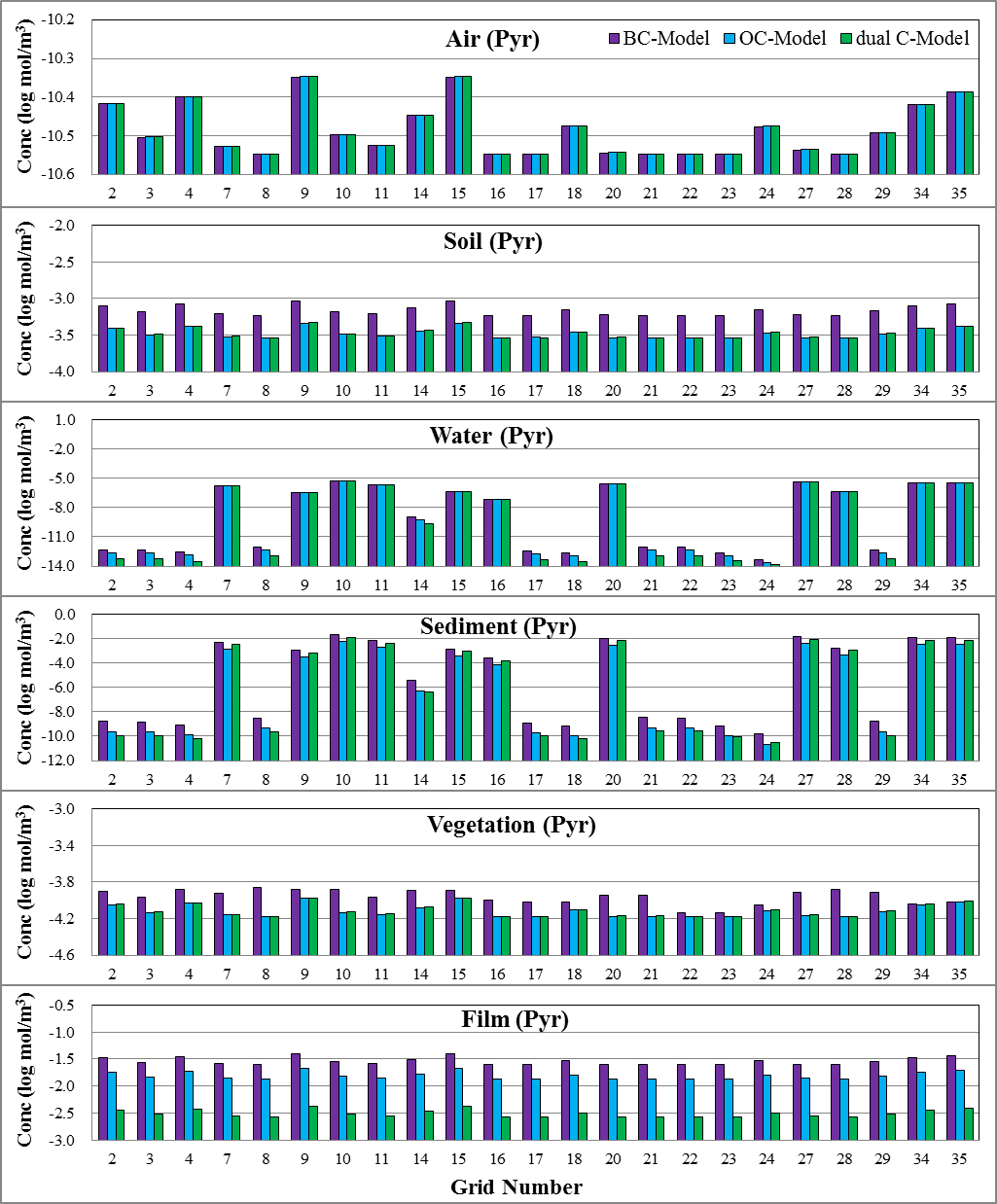


Figure S2


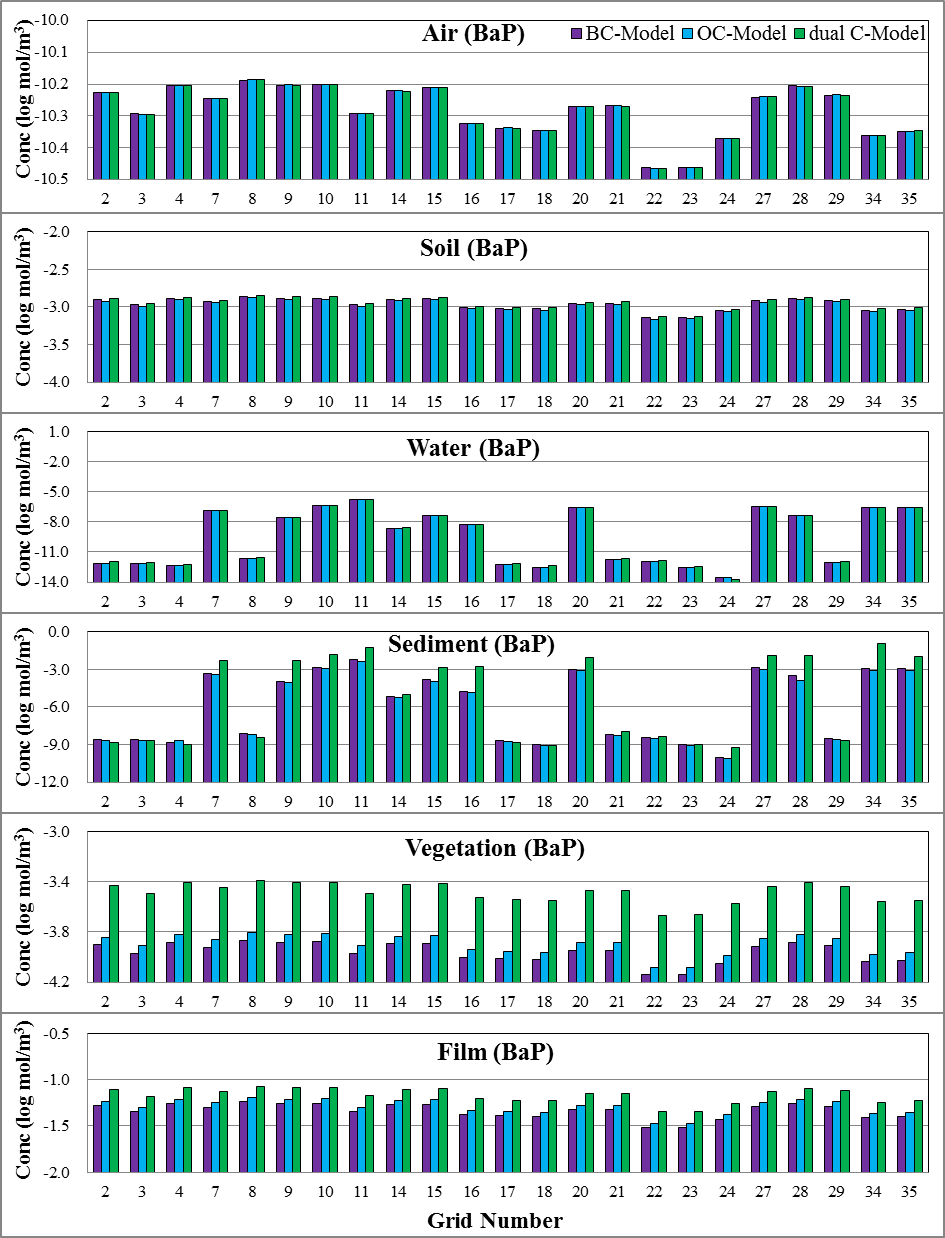


Figure S3


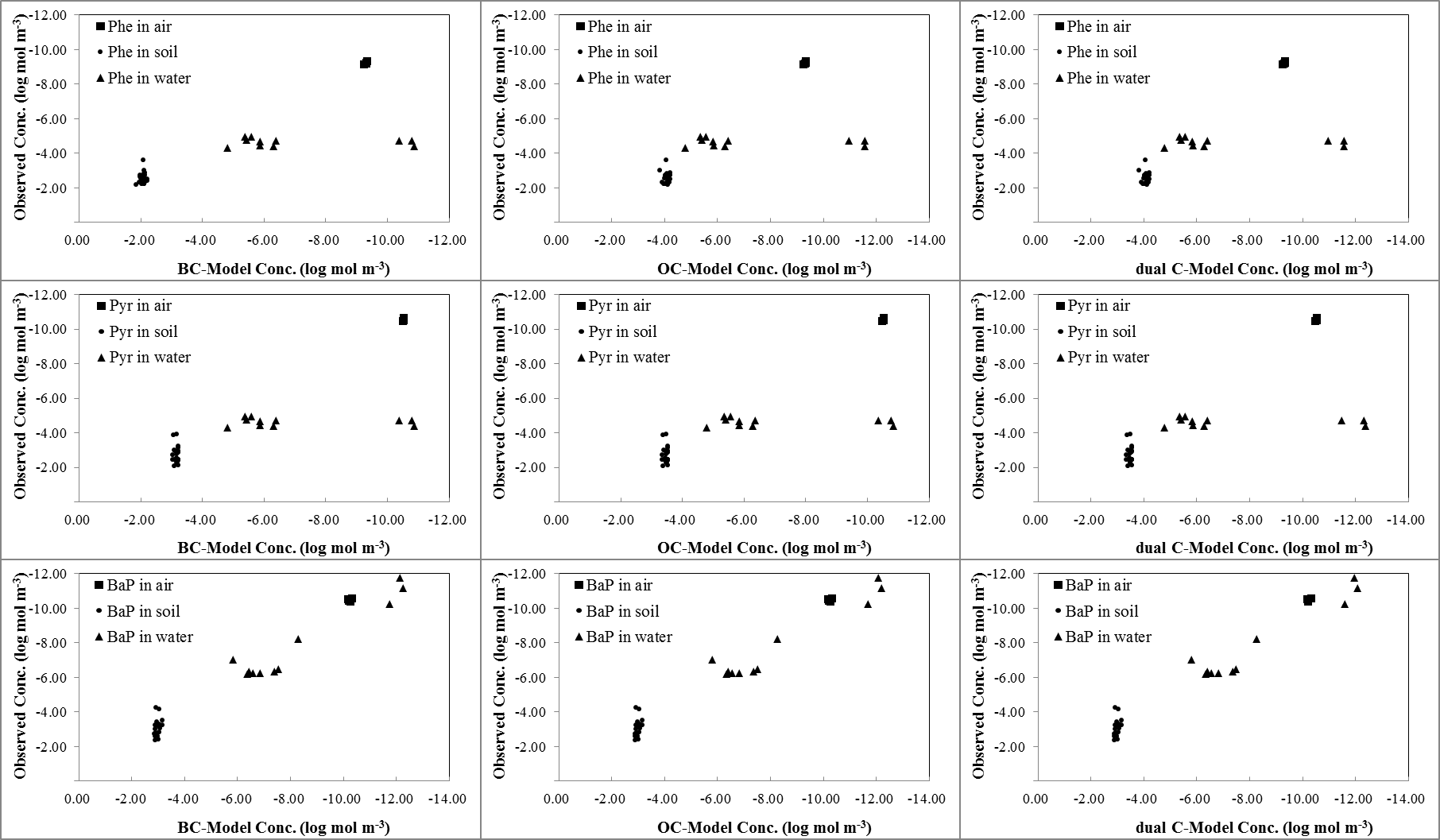


Figure S4


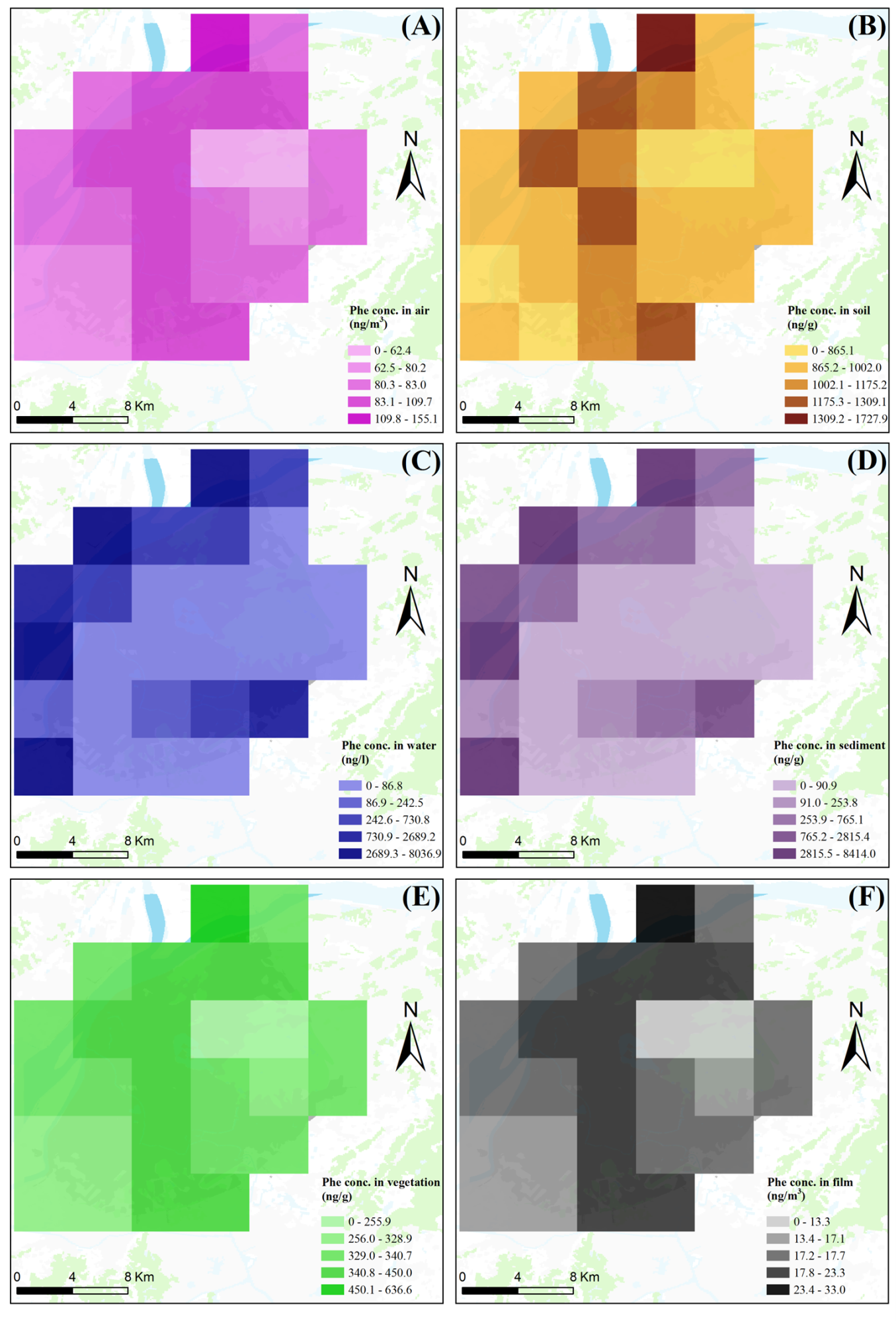


Figure S5


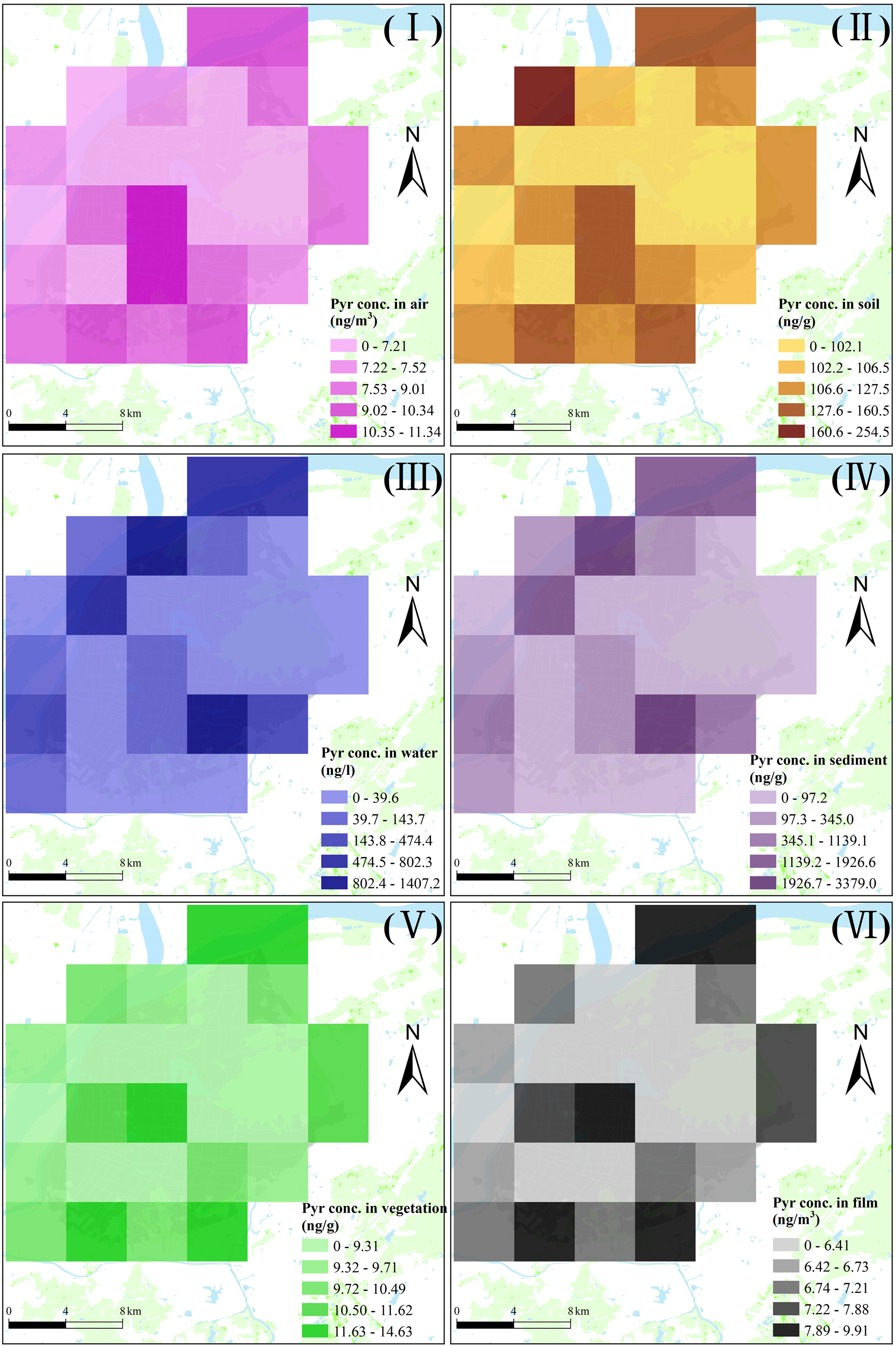


Figure S6


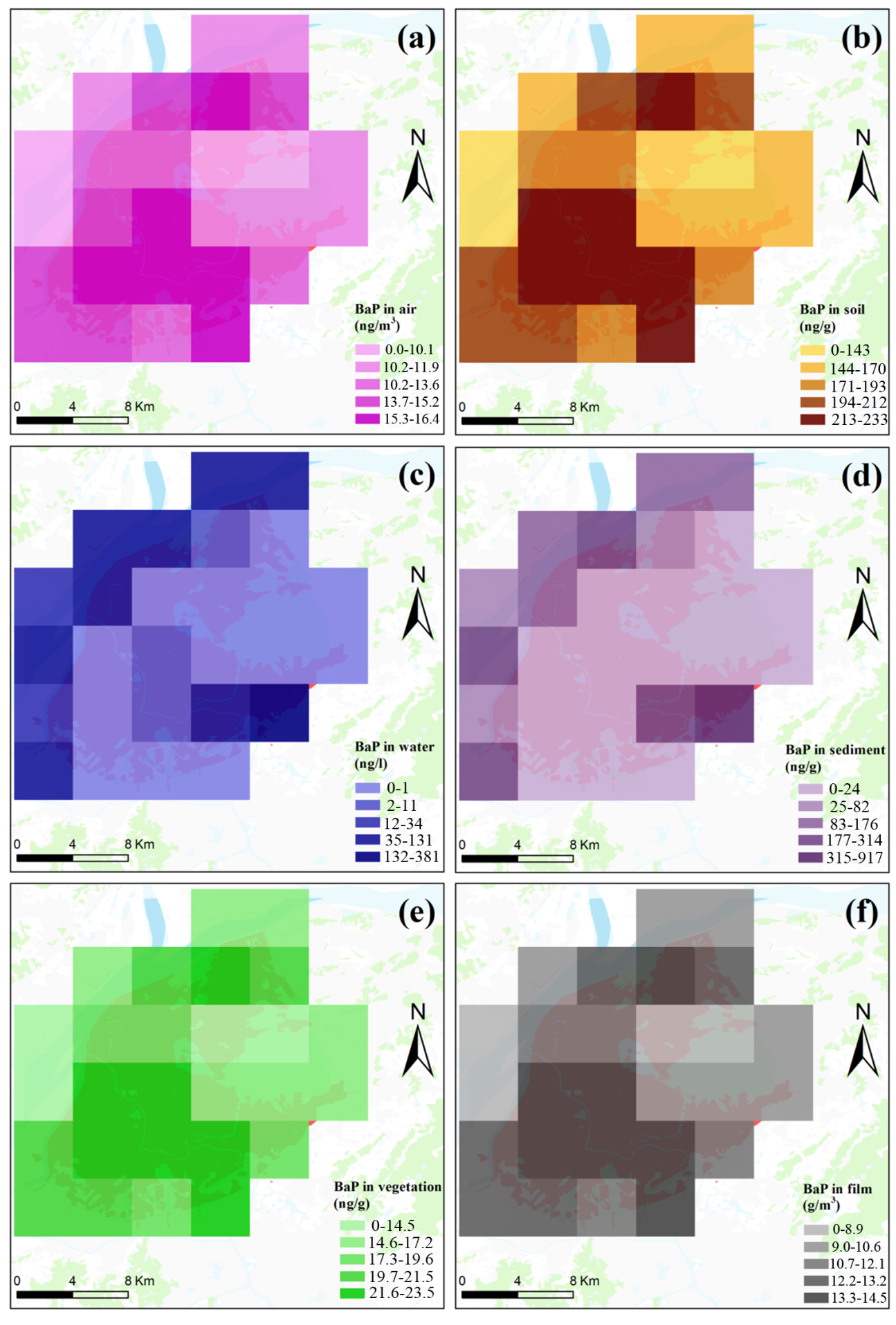


Figure S7

**References**

1. Riederer, M. Partitioning and transport of organic chemicals between the atmospheric environment and leaves in *Plant contamination: modeling and simulation of organic chemical processes* (ed. Trapp, S. & Mcfarlane, J.) 153-190 (1995).

2. Diamond, M. *et al*. Developing a multimedia model of chemical dynamics in an urban area. *Chemosphere* **44**, 1655–1667 (2001).

3. Paterson, S. & Mackay, D. Interpreting chemical partitioning in soil-plant-air systems with a fugacity model in *Plant Contamination, Modelling and Simulation of Organic Chemical Processes* (ed. Trapp, S. & Mcfarlane, J.) 191-214 (1995).

4. Cotham. W. & Bidleman, T. Polycyclic aromatic hydrocarbons and polychlorinated biphenyls in air at an urban and a rural site near Lake Michigan. *Environ Sci Technol* **29**, 2782-2789 (1995).

5. Tian, J. *et al*. Study on concentration and radiative forcing of black carbon aerosol in suburban Nanjing. *Climatic and Environmental Research (in Chinese)* **18**, 662-670 (2013).

6. Law, N. A preliminary multi-media model to estimate contaminant fate in an urban watershed. Masters Thesis, Department of Geography, University of Toronto (1996).

7. Huang, L. & Zhang, G. Seasonal variations of black carbon in Xuliujing water of the Yangtze River and their environmental implications. *Resources and Environment in the Yangtze Basin (in Chinese)* **23**, 1202-1207 (2014).

8. Huang, L. & Zhang, G. Distribution of black carbon in the sediments from the Yangtze River and their correlations with polycyclic aromatic hydrocarbons. *Earth and Environment (in Chinese)* **43**, 159-166 (2015).

9. Prevedouros, K., Palm-Cousins, A., Gustafsson, Ö., & Cousins, I. T. Development of a black carbon-inclusive multi-media model: application for PAHs in Stockholm. *Chemosphere* **70**, 607-615 (2008).

10.  Mackay, D. *Multimedia environmental models :the fugacity approach* (2001).

11. NSB. *Statistical yearbook of Nanjing* (2014).

12. Xie, Y. The emission characterization of PAHs in urban atmosphere and multimedia fate simulation in Shanghai. Thesis for Master Degree, Department of Geography, East China Normal University (2009).

13. NWCB. *Nanjing city water resources bulletin* (2014).

14. Yao, S., Li, S., Liu, J., Xue, B., & Xia, W. Recent sedimention of the core in southern Taihu lake inferred from ^137^Cs and ^210^Pb measurements. *Marine Geology & Quaternary Geology (in Chinese)* **26**, 79-83 (2006).

15. Hu, C. *et al*. Sediment resuspension observation in Taihu lake*. Science Bulletin* **50**, 2541-2545 (2006).

16. Bennett D., Mckone T., Matthies M., & Kastenberg W. General formulation of characteristic travel distance for semivolatile organic chemicals in a multimedia environment. *Environ Sci Technol* **32**, 4023-4030 (1998).

17. Ontario Ministry of the Environment (OME). *Metropolitan Toronto waterfront wet weather outfall study-phase I* 1995.

18. Bao, W., He, B., Bao, W. & Ding, D. Review on rainfall interception researches of forest vegetation. *Research of Soil and Water Conservation (in Chinese)* **11**, 193-197 (2014).
